# Novel gRNA design pipeline to develop broad-spectrum CRISPR/Cas9 gRNAs for safe targeting of the HIV-1 quasispecies in patients

**DOI:** 10.1038/s41598-019-52353-9

**Published:** 2019-11-19

**Authors:** Neil T. Sullivan, Will Dampier, Cheng-Han Chung, Alexander G. Allen, Andrew Atkins, Vanessa Pirrone, Greg Homan, Shendra Passic, Jean Williams, Wen Zhong, Katherine Kercher, Mathew Desimone, Luna Li, Gregory C. Antell, Joshua Chang Mell, Garth D. Ehrlich, Zsofia Szep, Jeffrey M. Jacobson, Michael R. Nonnemacher, Brian Wigdahl

**Affiliations:** 1 Department of Microbiology and Immunology, Drexel University College of Medicine, Philadelphia, PA 19102 USA; 2 Center for Molecular Virology and Translational Neuroscience, Institute for Molecular Medicine and Infectious Disease, Drexel University College of Medicine, Philadelphia, PA 19102 USA; 3 School of Biomedical Engineering and Health Systems, Drexel University, Philadelphia, PA USA; 4 Center for Genomic Sciences, Institute for Molecular Medicine and Infectious Disease, Drexel University College of Medicine, Philadelphia, 19102 Pennsylvania USA; 5 Center for Advanced Microbial Processing, Institute for Molecular Medicine and Infectious Disease, Drexel University College of Medicine, Philadelphia, 19102 Pennsylvania USA; 6 Sidney Kimmel Cancer Center, Thomas Jefferson University, Philadelphia, PA USA; 7 Department of Otolaryngology – Head and Neck Surgery, Drexel University College of Medicine, Philadelphia, 19102 PA USA; 8 Center for Clinical and Translational Medicine, Institute for Molecular Medicine and Infectious Disease, Philadelphia, PA USA; 9 Division of Infectious Disease and HIV Medicine, Department of Medicine, Drexel University College of Medicine, Philadelphia, PA 19102 USA; 10 Department of Neuroscience and Comprehensive NeuroAIDS Center, Lewis Katz School of Medicine, Temple University, Philadelphia, 19140 PA USA; 11 Department of Medicine, Section of Infectious Disease, Lewis Katz School of Medicine, Temple University, Philadelphia, 19140 PA USA; 12 Center for Translational AIDS Research, Lewis Katz School of Medicine, Temple University, Philadelphia, 19140 PA USA

**Keywords:** Computational models, Retrovirus

## Abstract

The CRISPR/Cas9 system has been proposed as a cure strategy for HIV. However, few published guide RNAs (gRNAs) are predicted to cleave the majority of HIV-1 viral quasispecies (vQS) observed within and among patients. We report the design of a novel pipeline to identify gRNAs that target HIV across a large number of infected individuals. Next generation sequencing (NGS) of LTRs from 269 HIV-1-infected samples in the Drexel CARES Cohort was used to select gRNAs with predicted broad-spectrum activity. *In silico*, D-LTR-P4-227913 (package of the top 4 gRNAs) accounted for all detectable genetic variation within the vQS of the 269 samples and the Los Alamos National Laboratory HIV database. *In silico* secondary structure analyses from NGS indicated extensive TAR stem-loop malformations predicted to inactivate proviral transcription, which was confirmed by reduced viral gene expression in TZM-bl or P4R5 cells. Similarly, a high sensitivity *in vitro* CRISPR/Cas9 cleavage assay showed that the top-ranked gRNA was the most effective at cleaving patient-derived HIV-1 LTRs from five patients. Furthermore, the D-LTR-P4-227913 was predicted to cleave a median of 96.1% of patient-derived sequences from other HIV subtypes. These results demonstrate that the gRNAs possess broad-spectrum cutting activity and could contribute to an HIV cure.

## Introduction

With the advent of highly active antiretroviral therapy (HAART), HIV-1 infection has become a manageable, chronic disease in many parts of the world, with the vast majority of treated individuals maintaining viral loads below the level of detection in clinical assays. While HAART is highly effective, it does not cure an individual, as it cannot clear the proviral DNA from within the latent reservoirs^1–5^. One of the hypothesized mechanisms of HIV-1 persistence is that the proviral DNA undergoes epigenetic modifications resulting in the repression of viral gene expression, ultimately leading to escape from immune surveillance. The most widely recognized latent reservoir is the resting memory CD4^+^ T-cell compartment^1,4–6^. This reservoir is very stable, with an estimated half-life of 44 months^4,6^. The quantitative viral outgrowth assay (Q-VOA) has been used to estimate that approximately one in a million CD4^+^ T cells harbor replication competent provirus at any given time point, while other assays that probe the latent reservoir by q-PCR have indicated that this number is closer to 300 in a million CD4^+^ T cells^7,8^, suggesting that most of the latent reservoir contains hypermutated and/or defective (or conditionally defective) provirus. Nevertheless, a significant portion may remain infectious but not reactivatable by traditional experimental methods. Studies performed by Ho *et al*. have shown that some proviruses cannot be reactivated even after multiple rounds of stimulation but are still fully infectious when reconstructed *in vitro*^9^.

Moreover, the latent reservoir includes cell and tissue compartments outside the resting memory CD4^+^ T cells like cells within the monocyte-macrophage lineage, cells within the gut-associated lymphoid tissue (GALT), and several types of cells within the central nervous system (CNS)^10–12^. As one important aspect of developing an HIV-1 cure using gene-editing-based technologies, it has therefore been necessary to consider proviral genetic diversity in all of these reservoirs, and not just within a single cell type or anatomical location. In addition, it has been shown that mutations accumulate in the proviral DNA during the conversion of the genomic viral RNA to DNA due to its error-prone reverse transcriptase and numerous host restriction factors. This results in each infected individual developing a spectrum of HIV-1 quasispecies (vQS) that is composed of large numbers of highly related viral variants. The vQS is a collection of viruses with a very similar genetic architecture but with genetically distinct sequences that exist within a patient. Every new mutation within a given genome is thought to result in a new vQS in that individual. Even within patients on stable and continuous HAART therapy, there is still low-level viral replication that allows for the continued drift and expansion of the vQS^2,13^. This low-level replication still allows for accumulation of and selection for viral single nucleotide polymorphisms (SNPs) that are functionally and clinically relevant to viral replication and pathogenesis as well as to clinical disease severity^14^.

The CRISPR/Cas9 technology has shown much promise for disrupting or altering the function of many genes, including viral genomes, in many different model systems^3,15^. Disruptive mutations result when the Cas9 enzyme induces targeted double-stranded DNA breaks, which are primarily repaired through the non-homologues end-joining (NHEJ) DNA repair pathway and result in insertions or deletions (InDels) at the target site. This technology has also been proven effective at inducing single base-pair changes, knocking in genes, knocking out genes, and regulating gene expression^15^. CRISPR/Cas9 has also been extensively investigated with respect to developing novel HIV-1 therapies, including: (a) prevention of HIV-1 infection of uninfected cells by altering vital cellular co-receptors, (b) activating latent provirus from within infected cells, (c) disruption of the proviral gene expression, and (d) excision of the entire provirus from latently infected cells^3,16–58^. Excision approaches have shown that the CRISPR/Cas9 system can delete the proviral genome from the host genome as well as induce hyper-mutation at the target site in a number of cell-based latency models and from *ex vivo* patient samples^24,29^. Similarly, HIV-1 transgenic rodent models and humanized mouse models have shown that CRISPR/Cas9 can reduce viral loads and excise viral genomes from cells in the peripheral blood, but most importantly also provirus in multiple other tissues and cellular reservoirs^28,49,53^. It is currently unclear whether recently implemented gRNAs targeting the LTR primarily act through an excision or by hyper-mutation of their targets. Research by Canver *et al*. has found an inverse relationship between the excision length and excision frequency; they estimate a 20% likelihood of excision of a 10Kb fragment and a 30% likelihood of excision of a 1Kb fragment^59^.

The main objective in tackling HIV-1 with CRISPR/Cas9-based strategies is to effectively reduce, if not eradicate, the HIV-1 proviral reservoir. Therefore, HIV-1 excision strategies have primarily targeted the proviral long terminal repeats (LTRs) that flank both ends of the proviral genome. In either case, the intrapatient and interpatient viral genetic variation within the LTRs present an obstacle for designing gRNAs for CRISPR-mediated therapies, since genetic variation in the target site could greatly reduce the efficiency of targeted proviral excision or mutagenesis. Previous research has shown that the majority of published anti-HIV-1 gRNAs target only a small fraction of patient-derived HIV-1 sequences even though some specifically targeted conserved, low entropy regions^19^. Some of these anti-HIV-1 gRNAs showed increased efficacy against multiple strains and subtypes, but they are still predicted to cleave only a small proportion of known patient-derived, subtype B sequences^19,42,43^. This is in part because available gRNA design tools are not constructed to compare a single gRNA against multiple target sequences simultaneously, nor to use multiple input sequences to make a broad-spectrum gRNA. Since the vQS is a continuously evolving target, multiple gRNAs with broad-spectrum activity will be absolutely necessary^3,19^.

The inherent promiscuity of gRNA-directed Cas9 activity presents both a challenge and an opportunity. Traditional applications of CRISPR/Cas9 avoid regions of genetic diversity and consider the promiscuity when screening for off-target activity^60^. However, as there are few positions in HIV-1 without genetic diversity, there is the potential to use the promiscuity to our advantage to target both common and rare variants. However, this requires sophisticated knowledge about how mismatches impact binding and cleavage efficiency. Research by Hsu *et al*.^60^ utilizing a collection of >700 gRNAs with intentional mismatches between the gRNA and target developed the first scoring algorithm for ranking off-target effects. A subsequent study by Doench *et al*.^61^ has improved on this algorithm by processing a larger collection of 27,897 gRNAs as well as incorporating both position- and nucleotide-specific mismatch penalties. These two techniques provide a way to quantify the likelihood of a cutting event given the pattern of mismatches between the gRNA and target.

Using these tools, we have developed an algorithmic pipeline that predicts panels of broad-spectrum gRNAs that collectively provide coverage for the above-mentioned proviral DNA heterogeneity in the HIV-1, subtype B vQS, while at the same time filtering out those gRNAs with any predictable off-target effects. Furthermore, the dbSNP database has been included in the analyses to more accurately account for the variation in the human genome. This analysis is an improvement over previous analyses in three primary ways. One, modern techniques are used to account for position- and nucleotide-specific mismatch effects between the gRNA and target DNA as opposed to *ad hoc* mismatch rules. Two, our computational analysis solely uses patient-derived sequence data as its template. Three, our pipeline has been able to quantify the likelihood of cleaving a vQS from deep sequencing data. We believe that this makes our analysis a first-in-class look at how to account for HIV genetic variation when designing broad-spectrum gRNAs.

## Results

### *In silico* testing of selected gRNA packages against multiple HIV-1-infected cohorts

Although several proposed anti-HIV-1 gRNAs have been shown to efficiently cleave their intended targets, few have been evaluated against the broad diversity of patient-derived HIV-1 proviral sequences. When all currently available anti-HIV-1 gRNAs were tested using an *in silico* algorithm against patient-derived, subtype B HIV sequences, many failed to be able to account for the extensive genetic variation observed within the vQS from sequences available in LANL, indicating that there was a need for broad-spectrum anti-HIV-1 gRNAs. This has been extensively reviewed in our previous publication^19^. Therefore, a gRNA design pipeline was devised to develop broad-spectrum anti-HIV-1 gRNAs for targeting the vQS in patients while simultaneously taking into account the natural genetic variation of the human genome, through incorporation of the dbSNP database, in order to further prevent the selection of gRNAs exhibiting off-target effects. In order to provide a set of diverse clinically-relevant proviral LTR sequences for the design of broad-spectrum gRNAs, LTRs from peripheral blood mononuclear cells (PBMCs) of 269 samples from 168 patients randomly selected from the Drexel CARES Cohort (Table 1) were amplified and deep-sequenced and supplemented with already sequenced samples from previous studies (Bioproject PRJNA309974). About half of the samples (57%) had undetectable viral loads at the sampled visit and most (73%) have no admitted history of drug use. We believe that using a patient dataset consisting of both well-suppressed patients and patients with readily detectable viral loads as well as across patients with and without drug use history allows us to examine the effect of gene-editing technology in clinically relevant contexts.

**Table 1:** Demographics of the subset of patients selected for LTR sequencing and gRNA design.

		269 Samples 168 Patients
Age		48 ± 7.1
History of Drug Use	Yes	26.5%
	No	73.5%
Gender	Male	73.5%
	Female	26.5%
CD4+ T cells (cells/mL)	Latest	503 ± 265
	Nadir	226 ± 187
Viral Load (copies/uL)	Latest	Undetectable (<100): 57%
		24,893 ± 79,255
	Peak	239,478 ± 555,838
Years Seropositive		16.55 ± 7.49
ART Status	On	98%
	Non-adherent	2%

**Figure 1:**
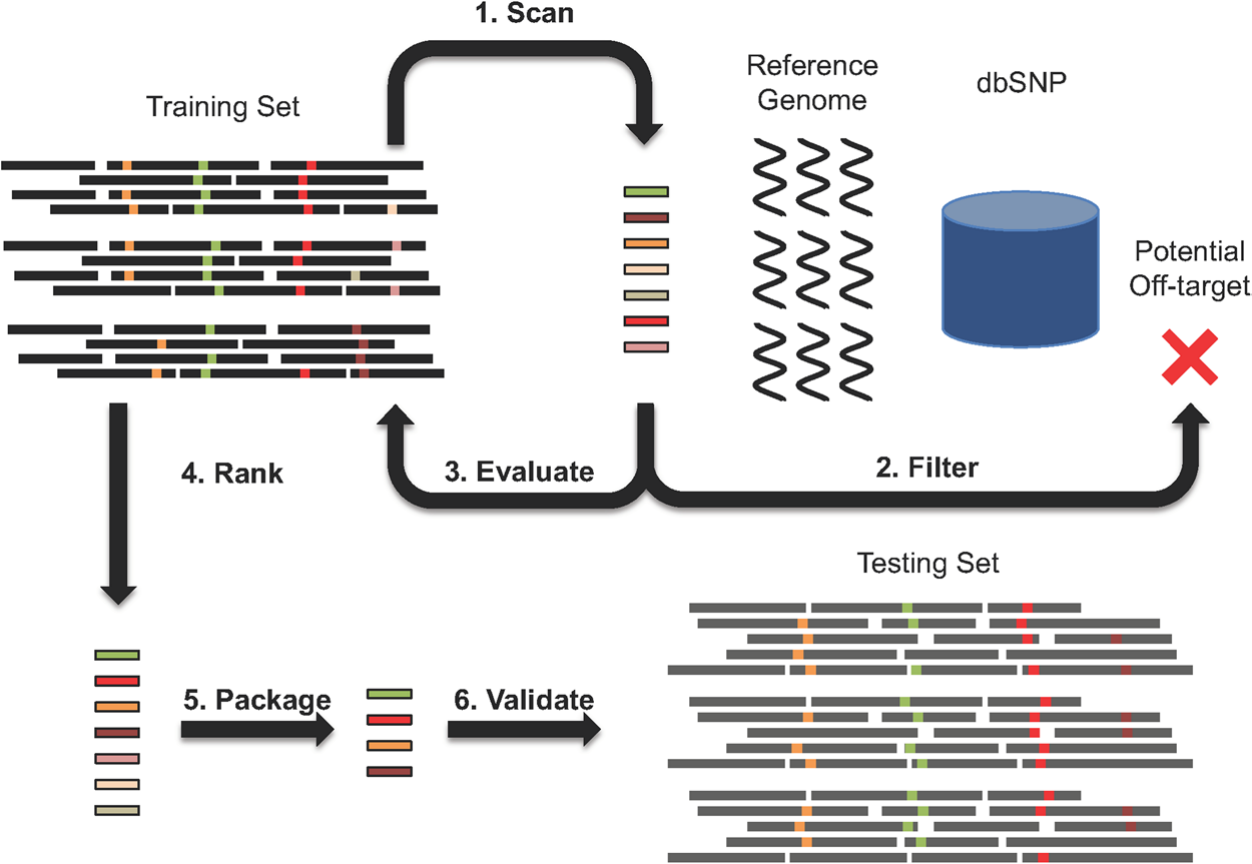
Workflow for CRISPR gRNA design. Whole blood was collected from a total of 269 HIV-1-infected samples from 168 patients enrolled in the Drexel CARES Cohort. Genomic DNA was isolated from PBMCs and a two round, nested PCR amplified the HIV-1 LTR as described in the Methods. These LTR amplicons were then deep-sequenced. The resulting sequence was then examined as follows for gRNA design: **(1)** The training set (100 samples) was scanned for all possible 20-mer protospacers; **(2)** an off-target search filtered out potentially dangerous gRNAs present in the human genome; **(3)** all remaining protospacers were evaluated against the training dataset to; **(4)** rank all gRNAs by their *in silico* efficiency; **(5)** package the top ranking gRNAs; and **(6)** validate the selected gRNAs against a held-out testing set (169 samples).

After sequencing, samples were randomly assigned to either a training dataset (100 samples) or a testing dataset (169 samples), care was taken to ensure samples from the same patient did not appear in both the training and testing datasets. After mapping the deep-sequenced reads to the HXB2 LTR, the sequences were processed using the pipeline described in detail in the Methods and in brief in Fig. 1. First, all unique PAM-adjacent 20-mer sequences found across all LTR sequence reads in the 100 sample training set were identified as potential treatment candidates. Next, an off-target search removed gRNAs present in the human reference genome or dbSNP build 144. The predicted cleavage efficiency was then calculated for the remaining gRNAs across the training set. After ranking the gRNAs, they were validated against the held out test set (169 samples). All gRNAs and packages of gRNAs were given a unique identifier based on the md5 hash of their sequence, this has been described in further detail in the Methods. When referring to all novel gRNAs developed here in this study and no one gRNA in particular, we will refer to them as Drexel gRNAs.

**Figure 2:**
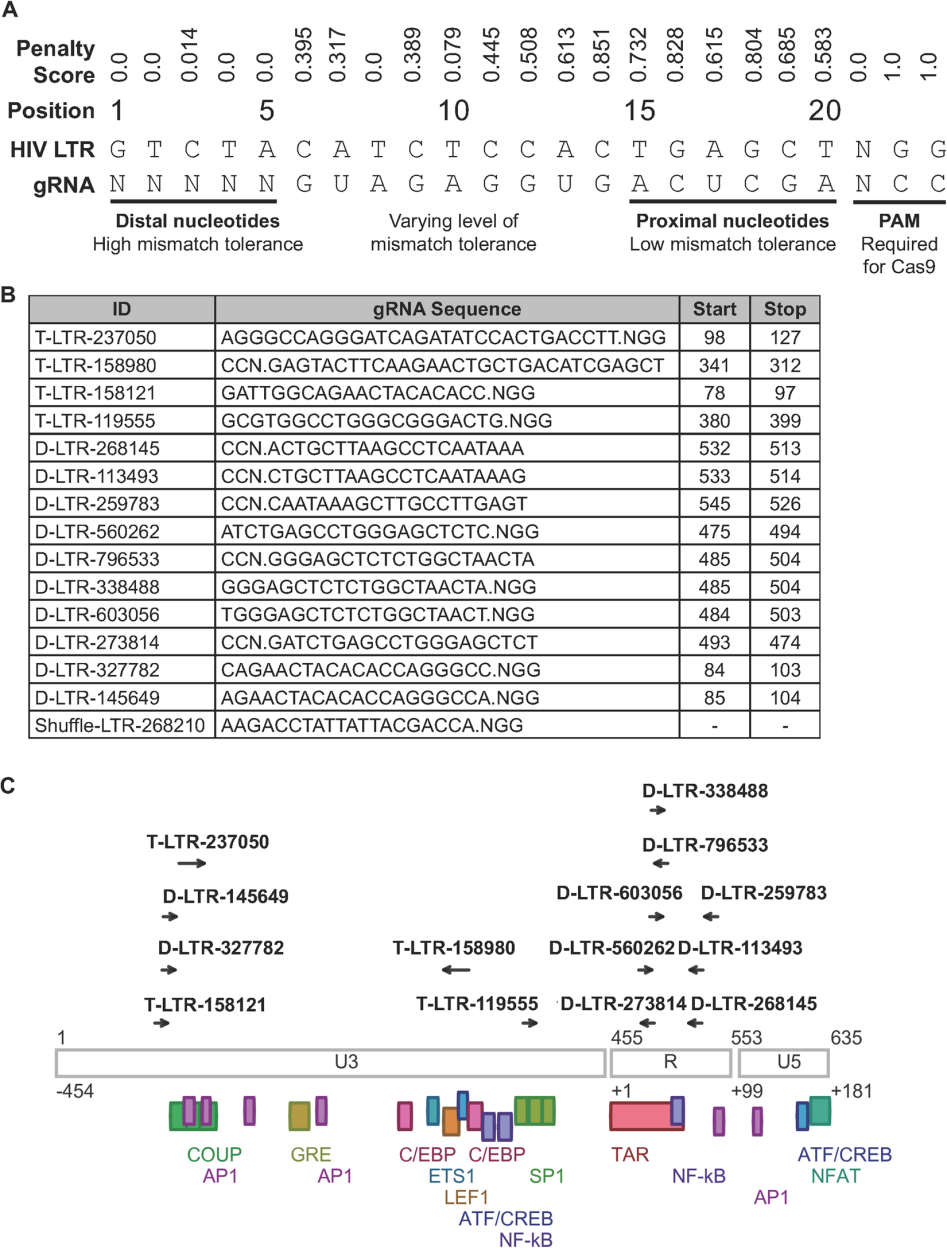
The distribution of Drexel gRNAs across the HIV-1 LTR. (**A**) The first 20 nucleotides of a gRNA bind to its complementary target sequence with variable tolerance to mismatches at different positions. Positions distal from the PAM have a higher tolerance to mismatches than proximal positions as indicated by the penalty score. The position-specific penalty score associated with mismatches between a gRNA and its target site was previously defined by Hsu *et al*. and others^19,60^. **(B)** The chart contains the sequence of the top 10 Drexel gRNAs (D-LTR) predicted to have broad-spectrum activity against diverse HIV-1 LTRs and their location within the HIV-1 HXB2 reference genome. The PAM indicates the direction of gRNA targeting. *Temple LTR-A, B, C, and D gRNA sequences (now referred to as T-LTR-237050, LTR-158980, LTR-158121 and LTR-119555) were obtained from Hu *et al*. for comparison^24^. Shuffle-LTR-268210 was used as a negative control. **(C)** The LTR schematic depicts the location of the Drexel gRNAs and the comparison gRNAs used in reference to the HXB2 LTR in relation to its structural features and common transcription factor binding sites.

The promiscuity of gRNA targeting, which allows imperfect complementarity to the target site, proved to be an advantage in gRNA design^3,19,60^, since positions distal to the protospacer adjacent motif (PAM) have a greater tolerance for sequence mismatches (as indicated by a low Penalty Score) in contrast to PAM-proximal positions (Fig. 2A**)**. However, no single gRNA was able to cleave 100% of samples. In order to effectively cover the vQS within and among individuals, sets of gRNAs were multiplexed as packages; we refer to the top 4 gRNAs and the top 10 gRNAs as D-LTR-P4-227913 and D-LTR-P10-287206, respectively. The gRNAs in each package primarily reside within the R region of the LTR, particularly within and around the trans-activation response (TAR) element **(**Fig. 2B,C). This was due to the high conservation of the area and low similarity to the human genome.

**Figure 3:**
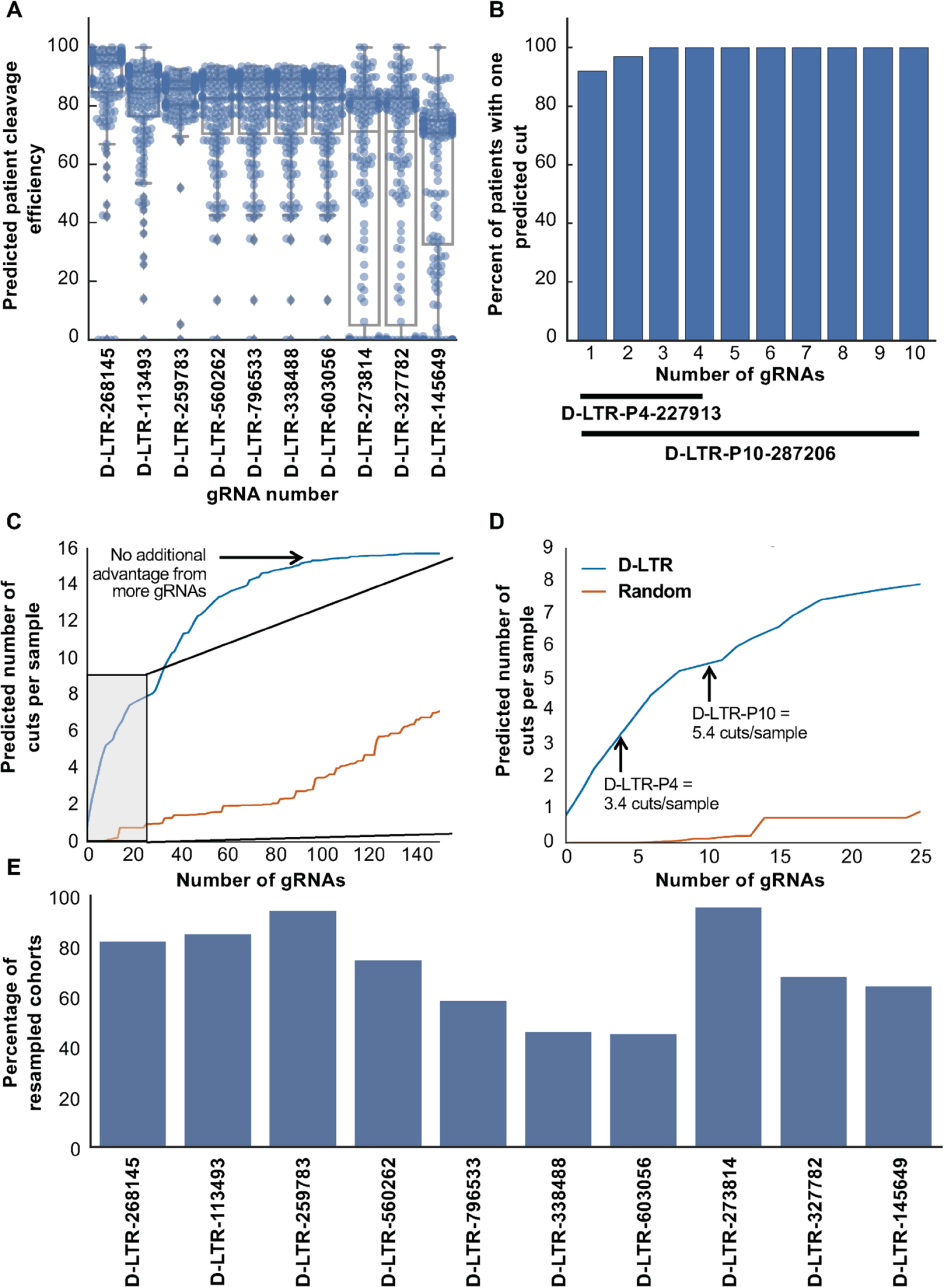
*In silico* predicted cleavage for the Drexel gRNAs predict 100% efficacy against patient-derived HIV-1 subtype B sequences from the held-out test cohort. (**A)** Depicts the predicted efficacy of each individual gRNA to cleave patient-derived HIV sequences. Each point represents the percentage of a patient’s vQS that are predicted to be cleaved by each gRNA for the 169 samples in the held-out cohort. The boxplots denote the quartiles, median and 95% confidence intervals. **(B)** Indicates the fraction 169 held-out samples with at least one gRNA predicted to cleave at least 70% of the sample. Column 1 represents the best performing gRNA as presented in **(A)**. Column two represents the combination of the best and second-best gRNA combined. D-LTR-P4-227913 and D-LTR-P10-287206 denote combinations of the top-4 and top-10 gRNAs respectively. **(C)** Depicts the number of predicted samples cut from the test cohort with an increasing number of gRNAs across the 169 held-out samples. A threshold is indicated where the number of efficacious gRNAs was maximized. The grey box is expanded in (**D)**. (**D**) An expanded view of the grey box from (**C**) demonstrates that D-LTR-P4-227913 and D-LTR-P10-287206 were predicted to specifically cleave more samples than by chance alone. **(E)** Indicates the percentage of times each gRNA was found to be in the top-10 best performing gRNAs in the validation cohort across 1000 iterations of randomly choosing 100 training samples and 169 testing samples.

To independently test the predicted effectiveness of the Drexel gRNAs against diverse proviral LTR sequences, they were tested *in silico* against all vQS identified by deep-sequencing analysis from the additional 169 test samples sequenced from the Drexel CARES Cohort. Individually, the top select gRNAs were predicted to effectively cleave the vQS in patients **(**Fig. 3A**)**. D-LTR-P4-227913 and D-LTR-P10-287206 were both predicted to cleave all of the LTR sequences from the 169 Drexel CARES samples in the test cohort **(**Fig. 3B**)**. Qualitatively similar results were seen when utilizing the Cutting Frequency Determination (CFD) matrix **(**Fig. S1A,B**)**. D-LTR-P4-227913 cleaved all patient sequences at least once with an average of 3.4 +/− 1.7 cuts per sample while the D-LTR-P10-287206 resulted in an average of 5.4 +/− 3 cuts across each quasispecies **(**Fig. 3C,D**)**. The training-testing procedure shows that our design pipeline selected gRNAs were predicted to target HIV-1 quasispecies from patient samples that it was not designed against. This was further validated through an iterative resampling procedure in which 1000 different iterations of the training and testing cohorts were devised and the top scoring gRNAs were determined for each of the iterations. The gRNAs that make up the D-LTR-P4-227913 were consistently found among the top-10 best performing gRNAs in at least 74% of the iterations **(**Fig. 3E). Qualitatively, similar results were found when evaluating patient cleavage ability with the CFD matrix **(**Fig. S1C**)**. The relationship between the number of gRNAs in the package and the average number of cuts per sample was further explored by extending this analysis to examine a range of gRNA package sizes ranging in size from 1 to 300 gRNAs. We identified a practical maximum of 16 cuts per sample by including roughly 100 gRNAs **(**Fig. 3C**)**. Coupled with the modest improvement between the D-LTR-P4-227913 and D-LTR-P10-287206, these data suggested diminishing returns to adding additional gRNAs to the package.

### gRNA cloning, transfection, and confirmation of Cas9 expression

The top four select Drexel gRNAs (D-LTR-268145, D-LTR-113493, D-LTR-259783 and D-LTR-560262) were each cloned and analyzed in a single donor vector that expressed the gRNAs under a Pol III promoter^24^. As it is important to compare the results of our designed package with a previously published set a collection of gRNAs, LTR-A through D were used and cloned into the same expression system (Addgene: 53186^62^) LTR-A through -D have now been referred to as T-LTR-237050, T-LTR-158980, T-LTR-158121 and T-LTR-119555 with their unique identifier. Since only the top three Drexel gRNAs were required to target all sequences tested *in silico*, D-LTR-268145, D-LTR-113493, D-LTR-259783 and D-LTR-560262 were individually cloned into expression vectors for initial experiments. The gRNA donor vectors were transfected into HEK293T cells and the HeLa-based HIV-1 reporter cell line, TZM-bl, to examine gRNA expression. Both cell lines were independently transfected with each of the four individual gRNAs and the Cas9 expression plasmid. Following a 24 hr expression period, the cells were harvested for isolation of protein and RNA. Cas9 protein was detected by western immunoblot analysis demonstrating the successful cloning and efficient expression of the individual gRNAs and the Cas9 enzyme **(**Fig. S2**)**. Similarly, using RT-PCR technology, the expression of all gRNAs in both cell lines was confirmed **(**Fig. S3**)**.

### CRISPR/Cas9 functionally disrupts LTR-driven transactivation and virus expression

**Figure 4:**
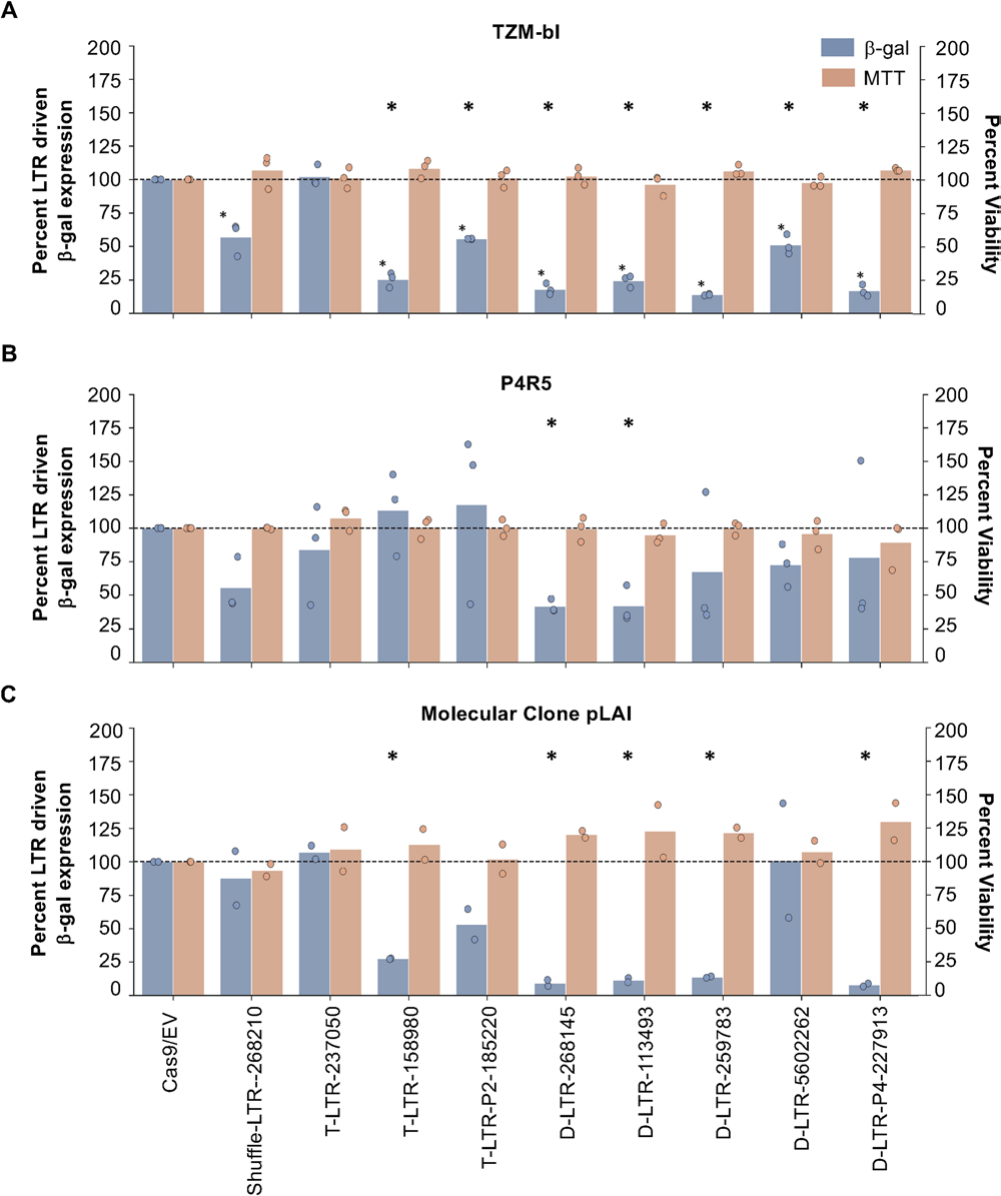
Treatment with Drexel gRNAs individually and together resulted in a significant reduction in LTR-driven transactivation. The HIV reporter cell lines TZM-bl **(A)** and P4R5 **(B)** were transfected with Cas9 and individual or packages of gRNAs concurrently with TatIIIB for 48 hr. Cells were then measured for viability (MTT, red bars) and reduction in LTR-driven ß-gal expression. In both cell types, Drexel gRNAs individually or together were able to effectively target and reduce LTR driven activity. **C)** P4R5 cells were transfected with Cas9 and different gRNAs concurrently with the fully infectious HIV-1 molecular clone pLAI. Cells were then measured for viability and reduction in LTR-driven ß-gal expression. Similarly, the Drexel gRNAs, individually or all together, were able to reduce viral gene expression and LTR driven expression through a number of proposed mechanisms. Statistical significance between Cas9/EV and experimental gRNAs was determined using a one-tailed, one-sample T-test and an *indicated p-values <0.05. Statistical significance between two gRNAs was determined by 2-tailed T-test comparing each item to the Cas9/EV samples. Each dot represents the average of four technical replicates.

By demonstrating successful cloning and expression of the CRISPR/Cas9 system, the next step was to demonstrate that the Drexel gRNAs were able to reduce LTR-driven viral gene expression. This was performed in two HIV-1 reporter cell lines, P4R5 and TZM-bl cells. Both cell lines possess an integrated HIV-1 LTR driving the expression of beta-galactosidase (β-gal) and/or luciferase; the LTR sequences in each of these cell lines have been shown to be clonal and as such are identical in all cells^24^. Several gRNAs were individually co-transfected along with HIV-1 strain IIIB Tat to activate the LTR. After 48 hr, LTR transactivation was measured by β-gal expression and compared to the Cas9/empty vector (EV) control. LTR-induced β-gal expression was not disrupted with Cas9/EV and showed no significant change. By contrast, β-gal expression was substantially and significantly reduced when treated with each of the four Drexel gRNAs in TZM-bl cells and with D-LTR-268145, D-LTR-113493 and D-LTR-259783 in P4R5 cells, above and beyond that observed for previously published T-LTR-237050 and T-LTR-158980 gRNAs **(**Fig. 4A,B). In both cell lines, there was a significant reduction in β-gal expression between T-LTR-237050 and D-LTR-268145 and D-LTR-113493 as well as between T-LTR-158980 and D-LTR-268145 in P4R5 alone. The substantial differences in gRNA-induced reduction in LTR-driven expression indicated a potential cell-type specific effect, possibly either due to proviral integration sites or differences in proviral-associated chromatin states. In addition, the lack of β-gal reduction from T-LTR-237050 and T-LTR-158980 might be the result of using Tat-mediated stimulation of the LTR instead of a more global promotor activator. Important to note is that T-LTR-237050 and T-LTR-158980 were not located within or around TAR like Drexel gRNAs. Importantly, β-gal reduction was not the result of cell death measured by the 3-(4,5-dimethylthiazol-2-yl)-2,5-diphenyltetrazolium bromide (MTT) viability assay in the presence of the CRISPR/Cas9 system **(**Fig. 4A,B**)**.

To further test whether Drexel gRNAs reduced expression driven by the integrated proviral DNA, P4R5 cells were transfected with the fully infectious, HIV-1 replication-competent molecular clone pLAI concurrently with the CRISPR/Cas9 system, and then β-gal measurements were performed to determine the level of CRISPR/Cas9-mediated disruption of HIV expression. In this assay, the pLAI produced infectious virus, and more importantly Tat which Drexel gRNAs that were co-transfected with Cas9 were able to potentially reduce β-gal expression in several ways: (a) induce mutations in the stably integrated P4R5 proviral LTR, (b) induce mutations in the pLAI plasmid LTR to reduce Tat expression, or (c) excision of the viral genome from pLAI. Drexel gRNAs were effective at disrupting β-gal expression, both singly and as a package, resulting in a 92% or greater reduction in LTR activity. When delivered as D-LTR-P4-227913, there was an even more significant reduction **(**Fig. 4C**)**. An MTT assay was performed to confirm CRISPR/Cas9 did not cause cellular toxicity compared to controls **(**Fig. 4C). Some differences among cell types were observed, for example D-LTR-560262 only showed a strong effect in TZM-bl and not in the other cell types. Similarly, the shuffled gRNA had no effect on β-gal expression in pLAI but did in P4R5 and TZM-bl cells. However, in all cases, the Drexel gRNAs reduced β-gal expression greater than either Cas9/EV and shuffled alone, indicating that reduced expression depends on Drexel gRNA targeting. Overall, these results demonstrated that the Drexel gRNAs individually or together as a package were directed to the LTR, induced InDels, disrupted Tat-mediated LTR transactivation, and inhibited viral gene expression.

### Drexel gRNAs severely alter the TAR stem-loop structure

**Figure 5:**
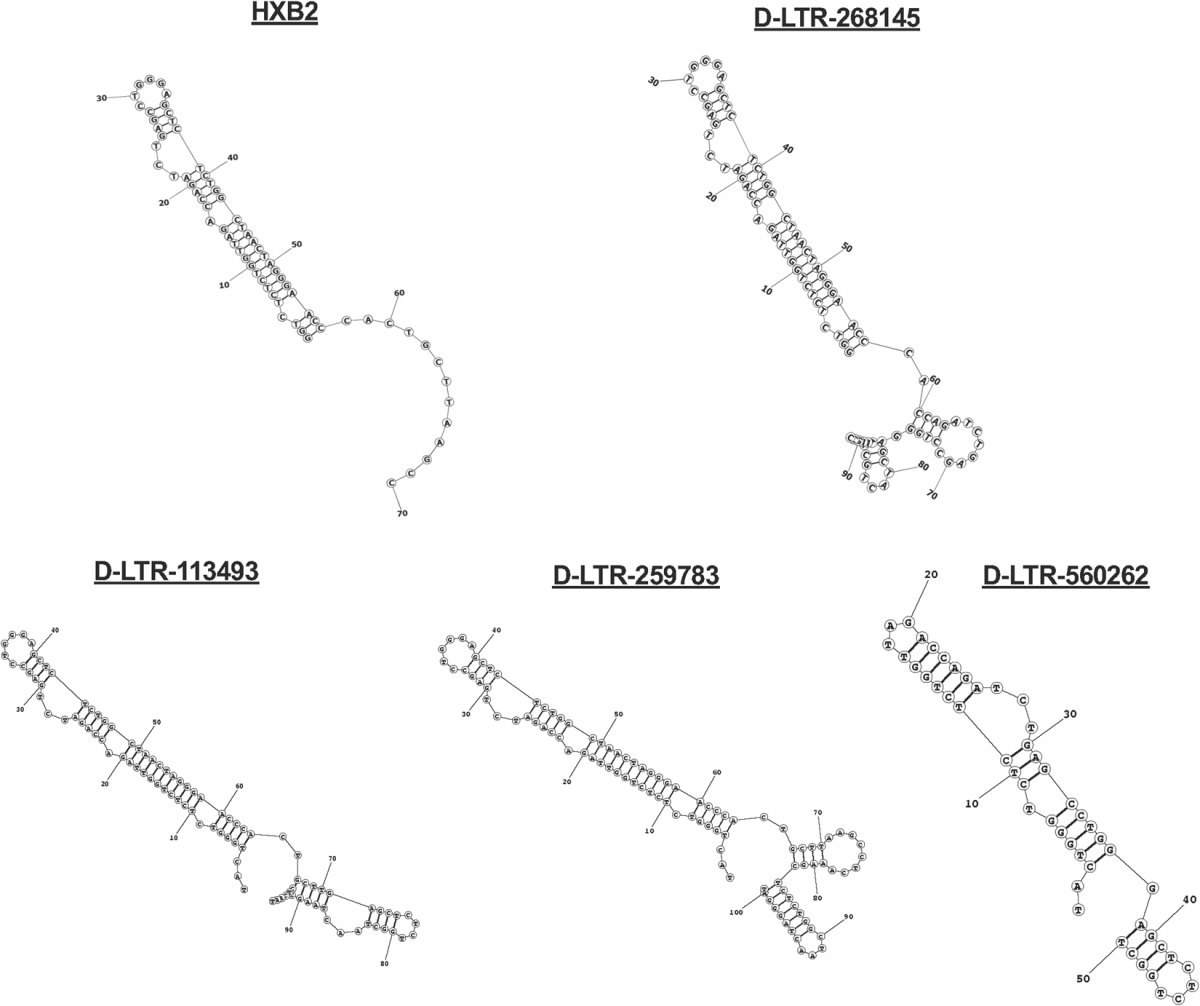
Drexel gRNAs target the LTR and alter the TAR stem-loop. TZM-bl cells were treated individually with D-LTR-268145, D-LTR-113493, D-LTR-259783 and D-LTR-560262. Next generation sequencing of LTRs was performed to determine the predicted secondary structure of the TAR stem-loop. Depicted here is a randomly selected secondary TAR structure for each Drexel gRNA in comparison to HXB2 TAR. See additional secondary TAR structures in Fig. S4.

Given that the Drexel gRNAs were highly effective at reducing LTR-driven β-gal expression compared to other gRNAs, it was hypothesized that this was due to disruption in the TAR secondary stem-loop structure given their complementary position in the LTR. Even small changes in the nucleotide sequence of the TAR stem-loop structure have been shown to have a profound impact on the secondary structure and thereby reduce Tat:TAR interactions and LTR transactivation, which are vital events in HIV-1 transcription and the overall process of viral replication^63,64^. To understand the functional impact of CRISPR/Cas9-induced InDels on the TAR structure, *in silico* secondary RNA predictions were performed from TZM-bl cells that were treated with the Drexel gRNAs, followed by next generation sequencing (NGS) analysis. Compared to the reference HXB2 TAR stem-loop, induced InDels profoundly disrupted the predicted TAR structure **(**Figs. 5 and S4). TAR structures for T-LTR-237050 treated samples were not analyzed, since its target site does not reside within the TAR region. These results demonstrate that D-LTR-268145, D-LTR-113493, D-LTR-259783 and D-LTR-560262 induce mutations within the LTR that disrupt the secondary structure of TAR and likely its transactivation ability **(**Fig. 4A–C**)** making them highly effective anti-HIV-1 inhibitors.

### D-LTR-268145 effectively cleaved the diverse vQS from patient-derived samples

**Figure 6:**
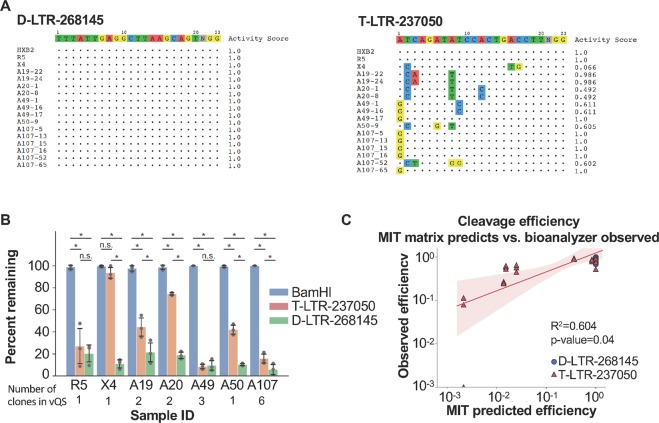
D-LTR-268145 can account for the genetic diversity of the vQS from patient-derived subtype B HIV sequences better than a previously published gRNA in a high sensitivity *in vitro* CRISPR/Cas9 cleavage assay. (**A)** LTR clones from Drexel CARES Cohort patients were amplified from PBMC genomic DNA, cloned, and sequenced. Mismatches between the D-LTR-268145 and T-LTR-237050 gRNA and the patient LTR target sites were aligned. The activity score indicated the predicted likelihood that the gRNA would cleave the target sequences with that particular mismatch or combination of mismatches. **(B)** *In vitro* CRISPR/Cas9 cleavage results of patient-derived HIV sequences representing the vQS. The number of clones in the vQS indicates the number of individual plasmids that were mixed in equal ratios. For example, patient sample A107 had 6 plasmid clones to make the vQS (clone A107-5, A107-13, A107-15, A107-16, A107-52 and A107-65) and mismatches within the target site for each clone was represented in subpanel A. Statistical significance was determined using Kolmogorov–Smirnov test and an * indicated p-values <0.05. (**C**) The scatter plot represents the correlation of the observed percent cleaved in the *in vitro* assay shown in B versus the predicted cleavage from the MIT activity score.

Although the Drexel gRNAs effectively targeted a single LTR sequence in a clonal population, evaluating whether these gRNAs would directly target the vQS required testing their efficacy at cleaving diverse patient-derived LTRs. To functionally validate the broad-spectrum activity of the Drexel gRNAs against the vQS and confirm their *in silico* efficacy **(**Fig. 3**)**, a high sensitivity *in vitro* CRISPR/Cas9 DNA cleavage assay was designed to directly assess patient-derived HIV-1 vQS cleavage. We previously cloned into pGL3 expression vectors diverse HIV-1 LTRs from the PBMCs of five patients enrolled in the Drexel CARES Cohort, selected based on differences within their HIV-1 LTR sequences compared to the T-LTR-237050 gRNA complementarity region^63,64^. D-LTR-268145 was selected from the pool of Drexel gRNAs as it had the best efficacy from *in silico* analysis and had the best response in all three assays presented in Fig. 4. T-LTR-237050 was used as a comparison as it has been efficacious in numerous assays in previous publications by Khalili and coworkers. The distinct LTR clones tested have been indicated in Fig. 6A, along with predicted activity scores of D-LTR-268145 and T-LTR-237050 against each target. To determine the cleavage efficiency against distinct targets *in vitro*, LTR clones derived from the same patient were mixed in equal ratios, incubated with an *in vitro* transcribed gRNA and recombinant Cas9, digested with BamHI to linearize untargeted plasmid, and reaction products were analyzed using a high sensitivity DNA chip on an Agilent Bioanalyzer (see Methods and Fig. S5). In all cases, both the D-LTR-268145 and T-LTR-237050 gRNAs cleaved the LTR clones, and in 4 of 5 patient samples (A19, A20, A50 and A107) the D-LTR-268145 gRNA was significantly more effective than the T-LTR-237050 gRNA **(**Figs. 6B and S6). D-LTR-268145 outperformed T-LTR-237050, because many of the LTR polymorphisms within the T-LTR-237050 target site, originally designed against a small collection of HIV-1 sequences, were in positions that had high penalties. This reduced cleavage efficiency when examining a larger cohort with T-LTR-237050. There was no observable CRISPR-induced cutting in the negative controls (Cas9 only, gRNA only, shuffled, or a gRNA targeting a host gene [RNF2]) **(**Fig. S6). These results support the ability of D-LTR-268145 to efficiently cleave the diversity found in the vQS of patient-derived HIV-1 sequences.

The LTR is not a static target and over the course of clinical disease, mutations accumulate even in the presence of highly suppressive antiretroviral therapy (albeit more slowly)^2,14^. In addition, changes in genotype can result in changes in clinical disease progression and presentation, as is the case with co-receptor utilization and the emergence of CXCR4 (X4)-utilizing virus from a predominantly CCR5 (R5)-utilizing vQS^65,66^. To demonstrate D-LTR-268145 can target distinct sequences relevant to changes in clinical disease presentation and accounting for viral variation over time, consensus X4 and R5 LTRs were determined from previously analyzed patient-derived subtype B, X4 and R5 HIV-1 LTR in the LANL and CARES Cohort databases^66^. As shown, the T-LTR-237050 gRNA and D-LTR-268145 gRNA can equally cleave the R5 LTR; however, the T-LTR-237050 gRNA cannot effectively cleave the X4 consensus LTR sequence in the *in vitro* cutting assay, while D-LTR-268145 gRNA can efficiently cleave the X4 consensus LTR **(**Fig. 6B). Again, this was due to a mismatch at position 16 and 17 which has high penalties associated with them. This result indicated that selected regions of the LTR may continue to evolve while the patient is on suppressive antiretroviral therapy and in some cases may become more resistant to targeting by a previously highly effective gRNA.

The results from the high-sensitivity *in vitro* CRISPR/Cas9 cleavage assays correlate with *in silico* predicted cleavage efficiency (Fig. 6C, r^2^ = 0.604 p < 0.05). This was most clearly demonstrated for patient A107, where our pipeline predicted five out of six clones would be cleaved by the T-LTR-237050 gRNA and all six by the D-LTR-268145 gRNA **(**Figs. 6B and S6). The agreement between the CFD matrix and the observed cleavage efficiency **(**Fig. S7**)** was confirmed and a markedly improved correlation (r^2^ = 0.984, p < 0.05) was obtained implying an increased accuracy. These results rigorously confirm the usefulness of both the MIT and CFD scoring matrices for optimizing gRNA design to accommodate proviral sequence variation.

### D-LTR-P4-227913 is predicted to be highly effective at targeting other HIV-1 subtypes

**Figure 7:**
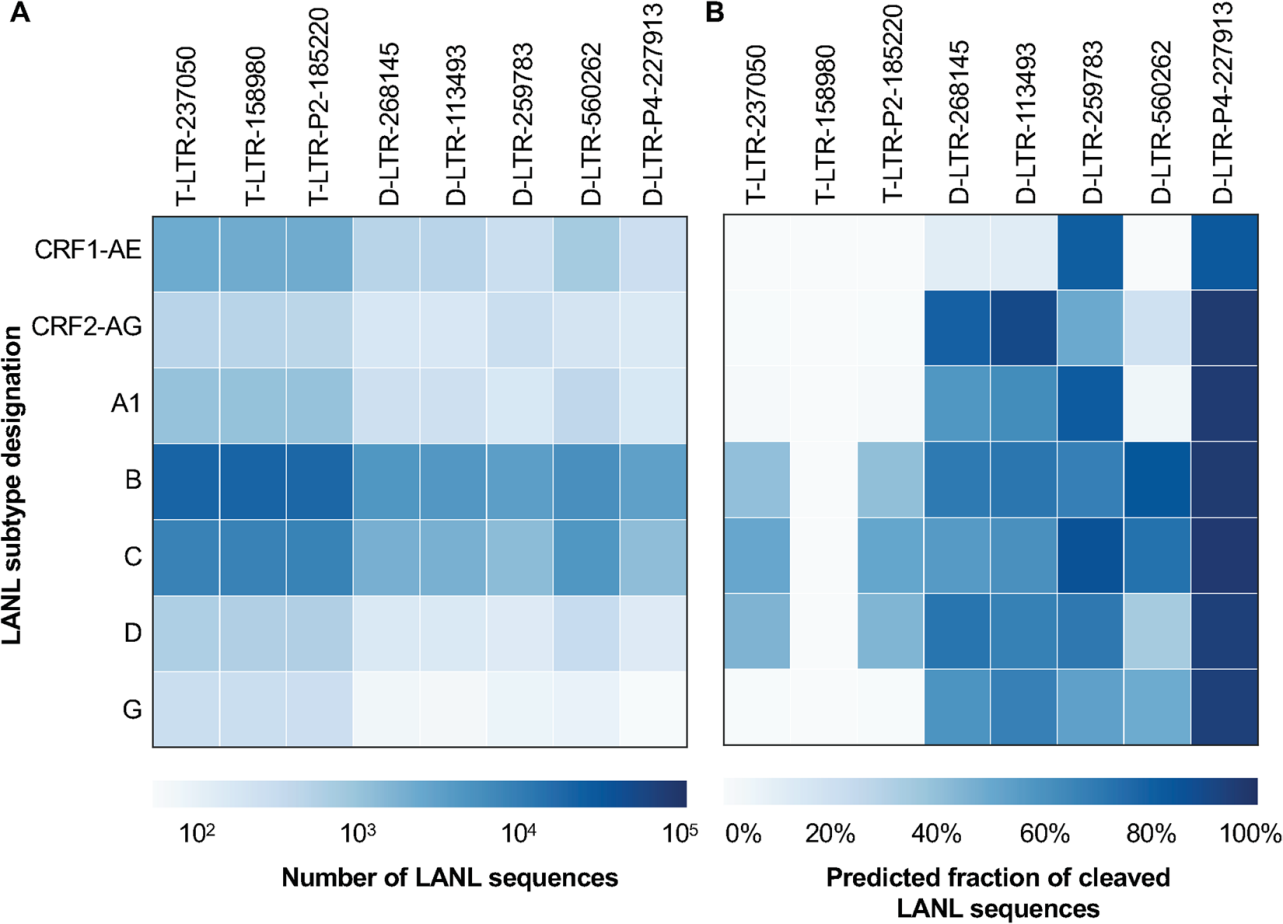
Drexel gRNAs are predicted to be highly effective at cleaving multiple HIV-1 subtypes *in silico*. (**A**) The number of patient-derived sequences from each subtype in the LANL database that overlap the gRNA binding site. Only those subtypes with at least 50 sequences across all binding sites are shown. (**B**) The percentage of patient-derived samples from each subtype that are predicted to be cleaved by the anti-HIV-1 gRNA. It is important to note that the gRNAs tested were designed using only subtype B sequences.

In an effort to understand how effective anti-HIV-1 gRNAs designed using subtype B sequences could be against other subtypes, they were tested *in silico* against a collection of sequences from multiple patient-derived HIV-1 subtypes in LANL. The fraction of patient-derived samples that could be cleaved from each subtype was calculated and has been shown in (Fig. 7A,B). This analysis showed that the Drexel gRNAs are predicted to be effective across a wide array of subtypes. Specifically, D-LTR-P4-227913 cleaved 100% of the subtype B sequences and a median of 96.1% of unique sequences from each common subtype with no subtype targeted with less than 82% effectiveness. This was surprising, since the Drexel gRNAs were only trained on subtype B sequences from the Drexel CARES cohort, and so this result indicated that there were highly conserved location(s) amongst patient-derived sequences across the HIV-1 subtypes. For T-LTR-237050 and T-LTR-158980, some subtypes were missed altogether or exhibited a low predicted cleavage. Collectively, these experiments demonstrate the broad-spectrum capabilities of the Drexel gRNAs in overcoming HIV-1 genetic diversity.

## Discussion

To apply the CRISPR/Cas9 system as a novel treatment for HIV-1 or as a prophylactic preventative agent, gRNAs will be needed that target HIV-1 sequences, in spite of intra- and inter-patient proviral genetic variation^3,19^. This is the first study to design and functionally test broad-spectrum anti-HIV-1 gRNAs at targeting both intra- and inter-patient sequence variation, showing that Drexel gRNAs target and cleave diverse vQS from patient-derived HIV-1 sequences. In this study, Drexel anti-HIV-1 LTR-targeting gRNAs were designed based on the results of patient-derived deep-sequencing data with the idea that such gRNAs could collectively cleave all the HIV-1, subtype B sequences within and among a large numbers of patients. This is vital because the vQS is not a static target within patients, even when their clinical disease and viral load are well-suppressed on HAART. We and others have shown that HIV-1 proviral DNA continues to evolve as detected in integrated HIV-1 provirus in the peripheral blood mononuclear cell (PBMC) compartment, albeit at much lower rates after initiating HAART^2,3,14^. This post-HAART mutation rate is not uniform across the HIV-1 genome and the LTR still displays moderate mutation rates^2,3^. It is important to consider this variation because the accumulation of mutations in the LTRs can impact clinical disease severity and functional viral fitness^2,3,14,19,63–66^. Therefore, designing broad-spectrum gRNAs that cover both cross-sectional and longitudinal patient-derived sequence variations is imperative for a CRISPR-based HIV-1 eradication program^3,19^.

In this study, deep-sequencing results from HIV-1-infected patients informed the design of broad-spectrum gRNAs. We have previously shown that only two published LTR-specific, anti-HIV-1 gRNAs were predicted to cleave >90% of patient-derived subtype B sequences from LANL^19^. This is in part because the target sequences do not represent the spectrum of variation within patient sequences and early gRNA designs were primarily intended for proof-of-concept in model systems that were designed specifically to knockout specific HIV-1 genes or regulatory elements to inhibit HIV-1 gene function or viral replication in general rather than for targeting a broad-spectrum of HIV-1 vQSs. Using patient-derived HIV-1 proviral deep-sequencing data along with a thorough algorithm design, the D-LTR-P4-227913 collectively target all of the viral genetic variation observed in the large number of patient samples examined in this study.

The efficacy of some of the Drexel gRNAs varied based on both the sequence of the gRNA and the HIV-1 reporter system. In P4R5 cells, approximately a 50% reduction was observed using D-LTR-268145 compared to an approximately 90% reduction in TZM-bl. When we compared these results with T-LTR-237050 and T-LTR-158980, we did not see this magnitude in reduction with either cell type. This is likely because of the unique location of the Drexel gRNAs, which resides within or proximal to the TAR encoding region. As evidenced from predicted TAR secondary structures, the Drexel gRNAs likely disrupt the TAR:Tat interaction, a highly specific RNA:protein interaction. We compared our results with those found by Mefferd *et al*.^67^ and Berkhout *et al*.^68^ who previously performed mutagenesis studies in the HIV-1 TAR region. Together, they found that there are many single mutations in the TAR structure that can ablate the TAR:Tat interaction. Taken together, these observations indicate that small mutations made within multiple parts of the Tar structure are enough to ablate Tat-mediated transcription.

On the other hand, the T-LTR-237050 and T-LTR-158980 gRNAs target the U3 region of the LTR, which has multiple transcription factor binding sites and redundancies. Therefore, the typical 3–7 base pair InDels induced from non-homologous end-joining by these gRNAs may not always disrupt LTR-driven transcription. The effectiveness of the Drexel gRNAs under Tat stimulation has implied that they disrupt this TAR:Tat interaction and that inactivating mutations in other regions might be overwhelmed in the presence of Tat. This is likely why there is no significant reduction in LTR-driven β-gal expression for some gRNA constructs. As Tat can be transmitted from cell-to-cell by exosomes^69^ and induce viral transcription in the recipient cell^70^ it is important to select gRNAs that will induce mutations that are effective even in the presence of Tat.

In addition, differences among gRNAs and cell lines with respect to reducing proviral expression may also be due to the form of stimulation used. In these experiments, TatIIIB was used to stimulate the LTR and β-gal expression. Previous literature has shown that closed chromatin can reduce gRNA binding and cleavage^71–74^. The LTR is surrounded by two nucleosomes that do encompass many of the gRNA target sites. This could, explain why T-LTR-237050 performed sub-optimally to T-LTR-158980, which does not reside within a nucleosome binding region. A more global activator such as PMA/TSA might more consistently displace nucleosomes around the LTR in P4R5 and TZM-bl cells, than the specific TatIIIB stimulation used here in a way similar to current “shock-and-kill” strategies.

A few studies have examined cleavage and rate of viral escape in the context of targeting coding regions versus non-coding region of the HIV-1 genome. From these studies, it was suggested that targeting coding regions of the HIV-1 genome was more effective at preventing viral escape than non-coding regions, which included targeting TAR^41–43,48,54,75^. This is because InDels in coding regions are more likely to disrupt necessary coding sequences and protein structure of essential viral proteins as opposed to non-coding regions. For the gRNAs targeting TAR, there was some virus escape, however, this was when a single gRNA targeting TAR was used. In general, when multiple gRNAs were multiplexed the likelihood for escape/resistance was reduced, as is the case with current retroviral therapy^34,41,42,54^.

Similar research by Mefferd *et al*.^67^ also examined gRNAs targeting the TAR region as well as Tat and the matrix and capsid regions of Gag. Upon close examination, the TAR1 gRNA studied has been shown to be identical, except for position 20 which has been shown to have no effect on efficiency of a gRNA, to the top-performing D-LTR-560262 gRNA. Their similar experiments in 293 T cells transfected with pNL-NLuc-HXB also showed a marked reduction in luciferase under unstimulated and Tat-stimulated conditions (Fig. 1)^67^. They further performed mutational resistance assays using stably transfected SupT1 cells expressing Cas9 and each gRNA of interest. In their experiments, only TAR1 prevented viral escape over a 32-day assay (Fig. 7)^67^. These independent results have provided evidence that targeting the Tar region will reduce the likelihood of viral escape.

Besides multiplexing our Drexel gRNAs in packages, we hypothesize the Drexel gRNAs were more effective than others because of their ability to target diverse patient sequences based on the inherit promiscuity of the gRNA itself as well as interfering with viral transcription/initiation and overall viral RNA stability. Downstream of the TAR stem-loop are other secondary RNA structure that are involved in other important aspect of the viral lifecycle. It could also be that Drexel gRNAs are disrupting the overall stability of the extensive secondary structure that exists downstream and thereby reduce one or more of the viral RNA transcripts. In addition, the simple occupancy of CRISPR/Cas9 on the TAR sequences may be disrupting transcriptional initiation and/or elongation because TAR stem-loop formation begins at the +1 transcription start site and continues to position +60. Others have suggested this possibility in studies using catalytically inactive Cas9 (dCas9) that was able to bind its target sequence but not cleave it. dCas9 binding alone within +1 to +100 of LTR R region was able to reduce virus expression^22,36,44,76^.

The high sensitivity *in vitro* CRISPR/Cas9 cleavage assay used here demonstrated that Drexel gRNAs efficiently cleave a mixture of diverse target sequences and that the *in silico* predicted gRNA efficacy correlated with *in vitro* cleavage **(**Fig. 6C**)**. Therefore, this *in vitro* cleavage assay can be used as a quick method to functionally analyze what fraction of target sequences would be cleaved by a particular gRNA and how well that correlates with predicted efficiency. This is especially important in the context of the continuously evolving LTR targets. We have previously demonstrated that gRNAs for some patients that were once effective at a particular point in time were not at a future point in time^3^. Now with a significantly larger sample size, the results here show that viral diversity representing longitudinal and cross-section sections can be targeted by the Drexel gRNAs. Due to time limitation and cost considerations, this is likely a better therapeutic approach rather than a fully personalized medicine approach; although this approach is also under investigation.

Treatment of D-LTR-268145, D-LTR-113493, D-LTR-259783 and D-LTR-560262 individually are highly effective at targeting the proviral LTR diversity, but together as a package (D-LTR-P4-227913) they are predicted to cleave sequences missed by any other published anti-HIV-1 treatment. This set of four gRNAs would fit within the current packaging ability of AAV and lentiviral vectors^53,62^. In this study, we have focused on designing LTR-specific gRNAs because InDels in the viral promoter can disrupt viral gene expression and because there are two identical copies of the LTR at either end of the viral genome, which can allow for excision of the entire provirus. Recently, it has been shown that a combination of anti-LTR and anti-Gag gRNAs have been effective^28,53^. This may be because the gRNAs are targeting both non-coding and coding regions of the viral genome, where if the provirus was not excised at least disruptive InDels were induced. In addition, while not functionally tested here, a D-LTR-P10-287206 and a Drexel package of 100 gRNAs were also designed. While this would not currently fit into a lentiviral- or AAV-based system, future advances in delivery may facilitate a larger delivery package. Therefore, other delivery methods such as nanoparticles or exosomes are being investigated due to their perceived ability to encompass a higher gRNA payload with the additional flexibility to enhance capabilities to deliver to specific target cell populations utilizing decorated nanoparticle structures^26,45,50^.

The analyses in this study demonstrate the effectiveness of the Drexel gRNAs against subtype B HIV-1 sequences but also showed that D-LTR-P4-227913 would be effective against most other HIV subtypes (Fig. 7). Our pipeline could be used to design gRNAs to more comprehensively target other HIV-1 subtypes, however, there is currently a dearth of sequences from non-B subtypes to allow for a proper train/test split. There are also no intra-patient NGS data from non-B or C subtypes publicly available, further limiting our ability to evaluate a pan-subtype anti-HIV-1 gRNA package. As data continues to be deposited it will be critical to ensure that this strategy will be applicable to all patients suffering from HIV infection.

A recent publication by Roychoudhury *et al*.^77^ has also explored the use of patient- derived sequences as the template for generating gRNAs with broad spectrum potential. They took a similar approach with the noted difference of using LANL data from four major subtypes as their initial training data and then used deep sequencing data for validation. In their search of the LTR region they also found all four members of D-LTR-P4-227913 as well as D-LTR-273814 and found comparable levels of coverage despite using a different scoring system. However, they explored only the packaging of up to three gRNAs and none of their proposed packages included any two of the D-LTR-P4-227913 members. These observations have emphasized the need for continued innovation in this research space.

A concern for all gene-editing strategies is the possibility of off-target effects. The Drexel gRNAs described here were screened against the human reference genome 19 (hg19), as well as including variation reported in the dbSNP database to also ensure no off-target effects at naturally occurring human SNPs. Because of this design, Drexel gRNAs are not predicted to have off-target binding to the human genome. This is important because recent studies have indicated that human genetic variation and more specifically SNPs can influence on- and off-target cleavage for gRNAs targeting host genes^78,79^. To ensure that there is no off-target excision and to confirm the *in silico* predictions presented here, an unbiased next generation sequencing technique such as Genome-wide Unbiased Identification of Double strand breaks (DSBs) Enabled by Sequencing (GUIDE-seq) and Comprehensive *In Vitro* Reporting of Cleavage Events by Sequencing (CIRCLE-seq) will be conducted in future experiments^80,81^. GUIDE-seq in particular will provide coverage on a genome-wide scale with next generation sequencing depth by identifying the insertion of a linker molecule at the sites of CRISPR-induced DSB. Genome-wide off-target discovery techniques are important in the process of gRNA design to avoid the trade-off between broad-spectrum anti-HIV-1 effectiveness and off-target effects. In addition, we have previously shown that when comparing thousands of virtual gRNAs there would be very limited binding activity with respect to endogenous retroviruses^82^. Overall, this study has demonstrated that the gRNA design pipeline we have developed is able to effectively design gRNAs targeting the vQS in infected individuals for the purpose of developing an excision-based HIV-1 therapeutic strategy.

## Materials and Methods

### Patient selection from Drexel CARES Cohort and sample processing

Experiments involving infected patient specimens and infection assays were performed in a BSL-3 facility. Patients enrolled in the Drexel CNS AIDS Research and Eradication Study (CARES) Cohort were recruited from the Partnership Comprehensive Care Practice of the Division of Infectious Disease and HIV Medicine in the Department of Medicine at Drexel University College of Medicine (Philadelphia, Pennsylvania, USA) and the Center for Clinical and Translational Medicine in the Drexel Institute for Molecular Medicine and Infectious Disease (Director, Dr. Zsofia Szep). Patients in the Drexel CARES Cohort were recruited under protocol 1201000748 (Brian Wigdahl, PI) approved by the Drexel University Institutional Review Board (IRB), which adheres to the ethical standards of the Helsinki Declaration (1964, amended most recently in 2008), which was developed by the World Medical Association as described^64^. All patients provided written informed consent upon enrollment. For this study, a subpopulation of 384 samples were randomly selected from the CARES cohort and subject to the following inclusion criteria: (a) had three or more visits available for analysis, (b) were currently on ART in their most recent visit, (c) had a last recorded CD4+ T-cell count ≥350 cells/ml, (d) the last VL recorded was ≤50, (e) had a visit within the last 12 months, (f) never had a comprehensive neurologic assessment that scored abnormal, (g) were not HCV-positive at any visit or (viii) had not tested positive for any drug of abuse at the last measured visit.

### Genomic DNA isolation and HIV-1 LTR PCR from patient samples and HIV-1 reporter cells

Genomic DNA (gDNA) and total RNA were isolated from patient PBMCs using the AllPrep DNA/RNA procedure (Qiagen). gDNA was isolated from HEK293T and TZM-bl cell lines using the QIAamp DNA mini procedure (Qiagen) 48 hr after transfection as previously described by the manufacturer. 125 ng of patient, HEK293T, or TZM-bl gDNA was utilized for a two-round, nested PCR using the high-fidelity Phusion polymerase for amplifying the HIV-1 LTRs. LTR primers for round one (LTR 3, 4) and round two (LTR 5, 6) were designed to optimally amplify patient-specific LTRs. Round one cycling conditions were as follows; 98 °C for 3 min, then 25 cycles at 98 °C for 10 sec, 50 °C for 20 sec, 72 °C for 18 sec, followed by a 10 min extension at 72 °C and a 4 °C hold. Either 2.5 or 10 μl from round one was used for the second round of PCR. Round two cycling conditions were; 98 °C for 3 min, then 30 cycles of 98 °C for 10 sec, 50 °C for 20 sec, 72 °C for 18 sec, followed by a 10 min extension at 72 °C and a 4 °C hold. Production of LTR products was confirmed by 1–2% agarose gel electrophoresis. Amplimers were sent for Sanger-based sequence confirmation using either LTR primers 5, 6 or 7 by GeneWiz. PCR products were either sent out uncleaned or following clean-up using either gel extracted PCR products, column cleanup or ExoZap. Sequences for LTR primers are provided in Table S1.

### Deep sequencing of patient-derived HIV-1 LTRs for Drexel gRNA design

Amplification of HIV-1 LTRs and deep-sequencing from patients enrolled in the Drexel CARES cohort was performed as previously described^2,14,63–65^. Briefly, patient PBMCs were isolated by ficoll gradient. Genomic DNA was isolated and an HIV-1 specific, two-round nested PCR was performed. The amplified LTR sequences were then purified and a library was tagmented using the Nextera XT Library Prep procedure with the Nextera XT Index procedure v2 to produce the sequencing libraries. Sequencing was performed on an Illumina NextSeq 500 instrument in paired-ended mode with a 300 High Output v2 sequencing procedure, as described by the manufacturer. This produced approximately 10 million reads per sample, providing the ability to query the diversity of the HIV-1 LTR vQS within each patient’s PBMCs. The sequences obtained from each patient sample were aligned to the HXB2 reference genome, Genbank accession K03455, for HIV-1 using the BWA mem, version 0.7.12 algorithm with default settings^83^. Using a cutoff of at least 1,000X depth across the LTR region, delimited by proviral sequence numbers 80–634, our analysis included a total of 269 samples. These samples were divided into a training cohort of 100 samples and an independent testing cohort of 169 samples. Iterative resampling of training and testing cohorts was performed by drawing 1000 repetitions randomly selecting 100 training samples and 169 testing samples, this uses the same cohort sizes as the original design. Final aligned sequences have been made available at the NCBI short-read archive (SRA), and all samples were linked under BioProject ID PRJNA309974.

### Design of gRNAs

Current web-portals, such as the MIT and CFD webservers, have been designed to extract potential gRNAs from a given input sequence and then scan a host genome for off-target effects. This has been considered to be insufficient for this analysis since the goal has been to understand how each potential gRNA will perform within the context of HIV-1 genetic variation. In order to address this limitation, the underlying statistical architecture behind the MIT and CFD webservers was reimplemented and encapsulated in the CRSeek Python package (https://github.com/DamLabResources/crseek). This package has provided a set of Python tools that facilitated the comparison of any user-supplied gRNA against any user supplied target DNA strand in a robust, repeatable, and highly scalable fashion (10.7287/peerj.preprints.27094v1). Two different penalty matrices were used to estimate the effect of a mismatch between the gRNA and the target DNA. The MIT penalty matrix assigns a position-specific penalty that is multiplied across all positions with a mismatch to the target followed by a penalty for highly mismatched targets. The CFD matrix assigns both a position- and nucleotide-specific penalty that is multiplied across all mismatches. Due to the ease of implementation allowed by the CRSeek package, all computational experiments were performed using both the CFD and MIT matrices.

The Drexel gRNAs were identified through a computational pipeline implemented using the CRSeek package by examining only the 100 samples randomly selected in the training set. NGS data from the patient specimens were aligned to the LTR region of the HXB2 genome (positions 1–750) using the bwa mem algorithm with default parameters^83^. Reads with a mapping quality greater than 20 were extracted and all potential protospacers were extracted and counted by extracting all sequences adjacent to an NGG PAM motif, on either strand. All unique protospacers were then re-scanned against the 100 samples by extracting all reads that overlapped the targeted position from each BAM file. The gRNA was then exhaustively compared to every position in the read if the maximum value exceeded 0.75 for the MIT matrix or 0.85 for the CFD matrix the read was assumed to be cleaved. The fraction of reads that overlapped the gRNA target which exceeded these cutoffs was used as a proxy for the percentage of the quasispecies that can be cleaved by a particular gRNA. These cutoffs were selected as they are the internal cutoffs used by the CFD and MIT webservers when marking sites as potential off-target hits.

The potential gRNAs were also screened for off-target effects across the human genome using a rigorous search technique. A local version of Cas-offinder was used to scan each protospacer against the human genome using a permissive 4 bp search. All potential hits were then intersected with the dbSNP build 144 to find all potential variants of these off-target sites across the known human variome. Any gRNA with any hit, either in the reference genome or a dbSNP variant, greater than 0.5 was excluded. This provided a list of gRNAs with a low likelihood of off-target effects across the known human variome. Hypothetically, this technique could be adapted to utilizing a personalized off-target profile by instead using a similar variant-called-format file that was generated from the patient’s own genome. However, as dbSNP already contains the majority of common SNPs in the population it is unclear whether this would improve the overall safety profile.

gRNAs that survived the off-target screening were ranked by their average cleavage efficiency across the training set. The top-10 gRNAs were formed into the D-LTR-P10-287206 and the top-4 were formed into the D-LTR-P4-227913. Using the same technique, the testing set of patient data (169 samples) was subjected to computational screening for cleavage efficiency. Packages of gRNAs were considered as independent cleavage events such that the likelihood of a set of gRNAs cleaving a patient was the complement of the product of the complement of their individual efficiencies. For example, a hypothetical package of two gRNAs with patient-specific efficiencies of 75% and 50% would have a combined efficiency of 1 − (1 − 0.75)*(1 − 0.5) = 0.875. Conceptually, this can be reasoned as: gRNA1 cuts 75% and leaves behind 25%; gRNA2 will, on average, cut 50% of what gRNA1 leaves behind resulting in a total of 87.5% of the vQS cut at least once with 37.5% being cut twice and only 12.5% missed.

As the gRNAs returned by this pipeline are dependent on the input dataset, we have devised a labeling scheme to track when the same gRNA is found across different runs of the algorithm. A number was generated from the MD5 hashsum of the protospacer and PAM sequence (NGG) **(**Fig. 2B and Table S2**)**. This 128-bit ID number will always be same across runs of the pipeline and across publications. For practical purposes only the 6 most significant digits are shown as this has not resulted in hash-collisions in practice.

### Analysis of historical HIV sequences

Sequences were downloaded from the Los Alamos National Laboratory HIV database in October 2015. Sequences shorter than 100 bp, having more than 10% ambiguous nucleotide composition, or were identified as being from a molecular clone were excluded. Sequences were limited to a single sequence per patient by grouping on the *PAT id(SSAM)* field and choosing the sequence from the lowest GI number. The subtype and geographic region designation provided by the database was used when grouping patients by either category. Using the databases annotations of HXB2, alignment positions sequences overlapping targeted regions, with at least 20 bp of sequence on each side, were extracted and tested for *in silico* cutting using the same method as described for the NGS sequences. When testing packages of gRNAs, the sequence was required to cover all targeted regions.

### gRNA cloning into the CRISPR expression vector

All gRNA expression plasmids (Catalog number 53186, 53187, 53188 and 53189) and Cas9 (Catalog number 41815) were purchased from Addgene. gRNA cloning was performed as previously described with some modifications^62^. Briefly, 20 bp forward and reverse gRNAs oligonucleotide were synthesized by Integrated DNA Technologies (IDT) with appropriate overhangs to be cloned into the specific gRNA backbone desired. After annealing, the gRNA was then phosphorylated for 1 hr in a 37 °C water bath followed by heat inactivation for 20 min at 65 °C. gRNA oligonucleotides were ran on a 1% agarose gel to determine their concentration for calculating the insert:vector molar ratio. gRNA expression vectors were cut with BbsI for 1 hr followed by heat inactivation for 20 min at 65 °C. In order to prevent the plasmid from ligation without the insert, the expression vector was dephosphorylated with shrimp alkaline phosphatase, incubated for 30 min at 37 °C and heat-inactivated for 15 min at 65 °C. A 10:1 insert to vector molar ratio was used for ligation of the insert into cut, dephosphorylated vector, for 2 hr at room temperature followed by transformation of 5 μl of the ligation mix into DH5alpha bacteria and grown overnight. Single bacterial clones were picked and grown for plasmid minipreps (Qiagen) and sequence confirmation of the gRNA inserts. M13 Reverse was used for mu6, hU6, 7SK, and H1 gRNA expression plasmid sequence confirmation of insert. Clones with the gRNA insert were maxipreped (Qiagen) and used for transfections.

### Cell lines and culturing conditions

TZM-bl cells (HeLa-based, human cervical epithelial cell line, transformed to have an HIV-1 LTR driving reporter constructs) were obtained from the AIDS reagent program and cultured in DMEM with 10% heat-inactivated FBS and penicillin (100units/mL)/streptomycin (100 µg/mL) as previously described^84–88^. 293 T/17 [HEK293T/17] (ATCC® CRL-11268TM) (human kidney epithelia cell line) cells were purchased from the American Type Culture Collection (ATCC) and cultured in DMEM, 10% heat-inactivated FBS, kanamycin sulfate and penicillin/streptomycin. P4 MAGI CCR5+ cells (P4R5) were obtained from the AIDS reagent program and cultured in DMEM, heat-inactivated FBS (10%), kanamycin sulfate, penicillin/streptomycin, sodium bicarbonate and puromycin (1 µg/ml)^89^. All cell lines tested negative for mycoplasma upon receiving them. In addition to being ideal for their intended assay, the cell lines that were selected for our analyses are widely available.

### Western immunoblot analysis

Transfected HEK293T or TZM-bl cells were lysed 24 hr post-transfection with RIPA buffer (Thermo). Protein lysates were quantitated using the Pierce BCA protein assay. Selected concentrations of protein lysates were subjected to SDS-PAGE on 12% Tris-glycine gels. Proteins were transferred to a PVDF membrane at 100 V for 55 min. The blot was blocked for 1 hr in 1X TBS, 0.05% Tween, and 3% nonfat dry milk. The blots were incubated overnight at 4 °C with either an anti-CRISPR-Cas9 antibody [7A9-3A3] HRP (ab202580) or a GAPDH antibody (Cell Signaling, #14C10). Primary antibodies were removed and the blot was washed three times for 10 min each with 1X TBS, 0.05% Tween, and 3% nonfat dry milk. The blot was then probed with a 1:10,000 dilution of anti-goat or anti-mouse IgG-IR conjugated (LICOR) antisera for 1 hr at room temperature. Secondary antibodies were then removed, and the blot was washed three times for 10 min each with 1X TBS, 0.05% Tween, and 3% nonfat dried milk, then the blot was wash three times for 5 min each with 1X TBS without milk. The blot was then visualized using an Odyssey imager.

### RNA isolation and RT-PCR

TZM-bl and HEK293T cells (5 × 10^5^) were seeded in a 6-well plates and transfected for 24 hr with each individual gRNA using lipofectamine 3000 as described by the manufacturer. RNA was isolated using the miRNeasy Mini RNA isolation procedure (Qiagen) and any contaminating DNA was removed by DNAse-I treatment and RNA cleanup (Qiagen). RNA (2000 ng) was reverse transcribed into cDNA using High Capacity cDNA Reverse Transcription reagent (Applied Biosystems). cDNA (5μl) was used for end-point PCR. GAPDH was used as an internal loading control. Cycling conditions were kept as previously described; 95 for 5 min, then 35 cycles of 95 °C for 30 sec, 55 °C for 30 sec, 68 °C for 30 sec, 68 °C for 5 min and 4 °C hold^62^. cDNA product (10 μl) was then run on a 2% agarose-ethidium bromide gel for UV analysis. Sequences for gRNA primers are provided in Table S1.

### Beta-galactosidase assay for LTR disruption

TZM-bl and P4R5 cells were seeded at 2.25 × 10^4^ cells in a 96-well microtiter plate and transfected with the CRISPR/Cas9 system and concurrently with the previously cloned pcDNA3.1+/hygro plasmid encoding the 86 amino acid TatIIIB protein (accession number, AAB59870)^63,64^. Cells were lysed 48 hr after transfection and analyzed using the Galacto-Star One-step B-galactosidase Reporter assay (Applied Biosystems). Lysate (10 µl) was incubated with substrate for 1 hr followed by reading on a GlowMax 96 dual injector plate reader (Promega). Three independent experiments each performed in quadruplicate for the TZM-bl and P4R5 β-gal knockdown studies. A similar experimental setup was used for experiments using the HIV-1 infectious molecular clone (pLAI) in P4R5 cells. pLAI was supplied by the AIDS reagent program^90^. For single gRNA transfections with pLAI, four independent experiments were performed in quadruplicate and 2 independent experiments in quadruplicate were performed for multiplexed gRNAs with pLAI. Error bars for all experiments show one standard deviation.

### MTT assay

To determine cell viability following transfection studies an MTT assay was performed. Briefly, following transfection of TZM-bl or P4R5 cells with CRISPR/Cas9, cultures were washed three times with PBS. Then complete media was added and incubated with 20 μl of 7.5 mg/ml thiazolyl blue tetrazolium bromide (Sigma) for 1.5 hr. Media was removed, and excess media was blotted off. Cells were lysed with acidic isopropanol for 20 min followed by analysis on a plate reader at 570 nm. At least three independent experiments in quadruplicate were performed for each condition except for multiplexed gRNAs with pLAI, which had two.

### *In silico* secondary RNA TAR stem-loop structure analysis

TZM-bl cells were transfected with 1,250 ng of Cas9 plasmid and 1,250 ng of each individual gRNA plasmid. Cells were stimulated 24 hr after transfection with 500 µl of PMA (250 nM)/TSA (250 nM) in complete media. gDNA was isolated 48 hr after transfection and a two-round, nested PCR was performed for subsequent deep-sequencing analysis as described in^14^. After alignment to HXB2, the reads that overlapped the target site were extracted and the exact sequence and placement of any indels were parsed from the CIGAR alignment string. Each unique InDel was cleanly inserted into an HXB2 backbone and the TAR region, HXB2:450–530 was extracted. The secondary structure of these sequences were predicted using the RNA Structure tool developed by Watson *et al*.^91^.

### *In vitro* Cas9 excision of the vQS

PMBC-derived patient LTRs from the Drexel CARES Cohort were amplified using the two-round nested PCR strategy. Patient-derived LTRs amplified from PBMCs were cloned into the pGL3 basic expression vector as previously described *(63, 64)*. X4 and R5 consensus LTRs were constructed from previous analyses^66^. These consensus LTRs were synthesized and cloned into the pGL3 vector by VectorBuilder (Cyagen Biosciences). gRNA oligonucleotides were purchased from IDT and were used for *in vitro* transcription using the EnGen sgRNA synthesis procedure (NEB) as previously describe by the manufacturer. Oligonucleotides for *in vitro* transcription of gRNA are provided in Table S1. *In vitro* transcribed gRNAs were purified and concentrated using the RNA clean and concentrator procedure (Zymo Research). The *in vitro* digestion of patient LTRs was performed following NEB *in vitro* digestion of DNA with Cas9 nuclease protocol with few modifications^92^. The patient-derived LTRs cloned into pGL3 were added in equal ratios. Cutting reactions were performed for 1 hr at 37 °C followed by 65 °C heat inactivation for 5 min. BamHI restriction digestion was performed to linearize any undigested plasmid and samples were cleaned using the Qiagen PCR cleanup procedure to ensure proper running on the High Sensitivity Bioanalyzer chip (Agilent). The sizes and molarities identified by the Bioanalyzer were used for the calculation of gRNA/Cas9-induced cleavage efficiency. The pGL3 expression vector is 5.5 kb long with the patient-derived LTR cloned within. As the sizes reported by the Bioanalyzer have a normal standard error and a mean shift clustering algorithm was used to identify the appropriate cutoffs to use when assigned a Bioanalyzer peak to a particular molecular fragment. We chose a width of 500 bp due to the rough estimate of 10% error in the length estimation provided by Agilent. The average size of the linearized pGL3 observed by the Bioanalyzer was 5,495.15 bp with a standard deviation of 329.75 bp after clustering. As a result, the peak call with a size between 5,055 and 6,604 bp was considered as full-size of pGL3 observed in Bioanalyzer in subsequent analyses. The two fragment sizes resulting from the Cas9 *in vitro* digestion were calculated in a similar way. The calculation of cleavage efficiency, the mean of the molarity of two Cas9-digested fragments (designated as *mol*_*f*1_ and *mol*_*f*2_ in Equation below) was used as the molarity for the fragmented pGL3. The molarity of the total pGL3 was calculated by adding the molarity of fragmented pGL3 and the molarity of unfragmented pGL3 (designated as *mol*_*un*_). The function of cleavage efficiency can be described as

$$cleavage\_efficiency=\frac{(mo{l}_{f1}+mo{l}_{f2})/2}{mo{l}_{un}+(mo{l}_{f1}+mo{l}_{f2})/2}$$

and the percentage of pGL3 remaining undigested by gRNA/Cas9 as:

$$percent\_remained=(1-cleavage\_efficiency)\times 100 \% $$

Statistical significance between gRNAs and controls was determined using the Kolmogorov- Smirnov (K-S) Test as the efficiency values are not normally distributed.

## Supplementary information

Supplementary Materials

## Data Availability

The data sets generated during and/or analyzed during the current study are available at the NCBI short-read archive (SRA), and all samples were linked under BioProject ID PRJNA309974. Sequences mapping to the human genome were removed before uploading. “The following reagent was obtained through the NIH AIDS Reagent Program, Division of AIDS, NIAID, NIH: TZM-bl from Dr. John C. Kappes, Dr. Xiaoyun Wu and Tranzyme Inc.”; P4.R5 MAGI from Dr. Nathaniel Landau.”; pLAI.2 from Dr. Keith Peden, courtesy of the MRC AIDS Directed Program.”

## Code availability

The code used in this manuscript is kept at https://github.com/DamLabResources/crseek. Figures and analyses were generated with version 13d8870.
