# Supplementary material for: Novel gRNA design pipeline to develop broad-spectrum CRISPR/Cas9 gRNAs for safe targeting of the HIV-1 quasispecies in patients
Source: Sci Rep. 2019 Nov 19;9:17088. doi: 10.1038/s41598-019-52353-9 (PMC6864089; doi:10.1038/s41598-019-52353-9)
Supplement: Supplementary file 1 — Supplementary Materials [file 41598_2019_52353_MOESM1_ESM.pdf]

Novel gRNA design pipeline to develop broad-spectrum CRISPR/Cas9 gRNAs for safe targeting of the HIV-1 quasispecies in patients

Authors:
Neil T. Sullivan[1,2,#], Will Dampier[1,2,3,#], Cheng-Han Chung[1,2], Alexander G. Allen [1,2], Andrew Atkins[1,2], Vanessa Pirrone[1,2], Greg Homan[1,2], Shendra Passic[1,2], Jean Williams[1,2], Wen Zhong[1,2], Katherine Kercher[1,2], Mathew Desimone[1,2,3], Luna Li[1,2], Greg Antell[1,2,3], Joshua Chang Mell[1,2,4,5], Garth D. Ehrlich[1,2,4,5,6,7], Zsofia Szep[8,9], Jeffrey M. Jacobson[10,11,12], Michael R. Nonnemacher[1,2,6], and Brian Wigdahl[1,2,6*]

Supplemental Materials:

Figure S1

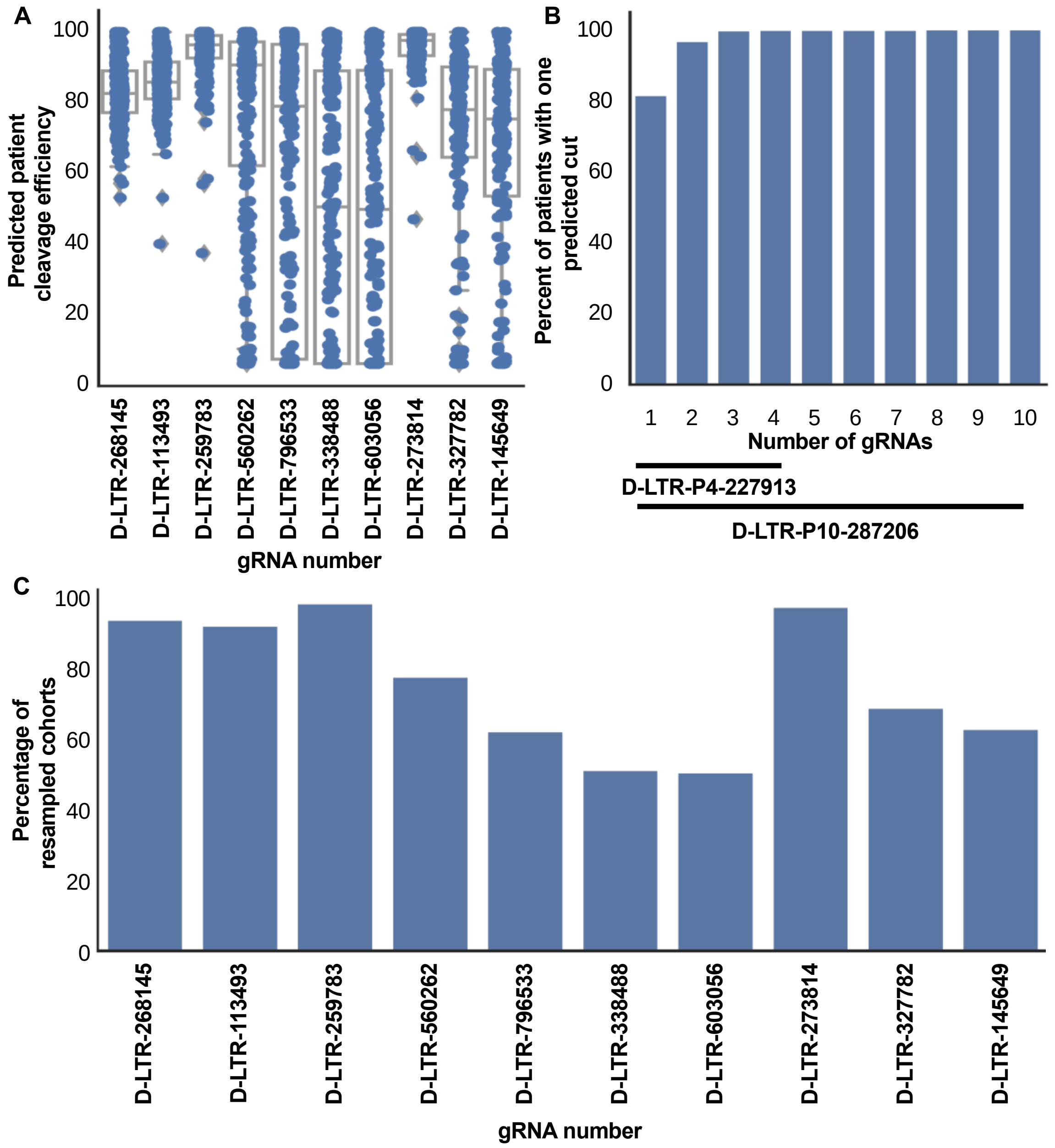

Figure S1: Validation of *in silico* predictions using the CFD matrix. (A) Depicts the predicted efficacy of each individual gRNA to cleave patient-derived HIV sequences. Each point represents the percentage of a patient's vQS that are predicted to be cleaved by each gRNA using the CFD matrix (n=169). The boxplots denote the quartiles, median and 95% confidence intervals. **(B)** Indicates the fraction of samples with at least one gRNA predicted to cleave at least 70% of the sample (n=169). Column 1 represents the best performing gRNA as presented in **(A)**. **(C)** Indicates the percentage of times each gRNA was found to be in the top-10 best performing gRNAs in the validation cohort across 1000 iterations of choosing 100 training samples and 169 testing samples.

Figure S2

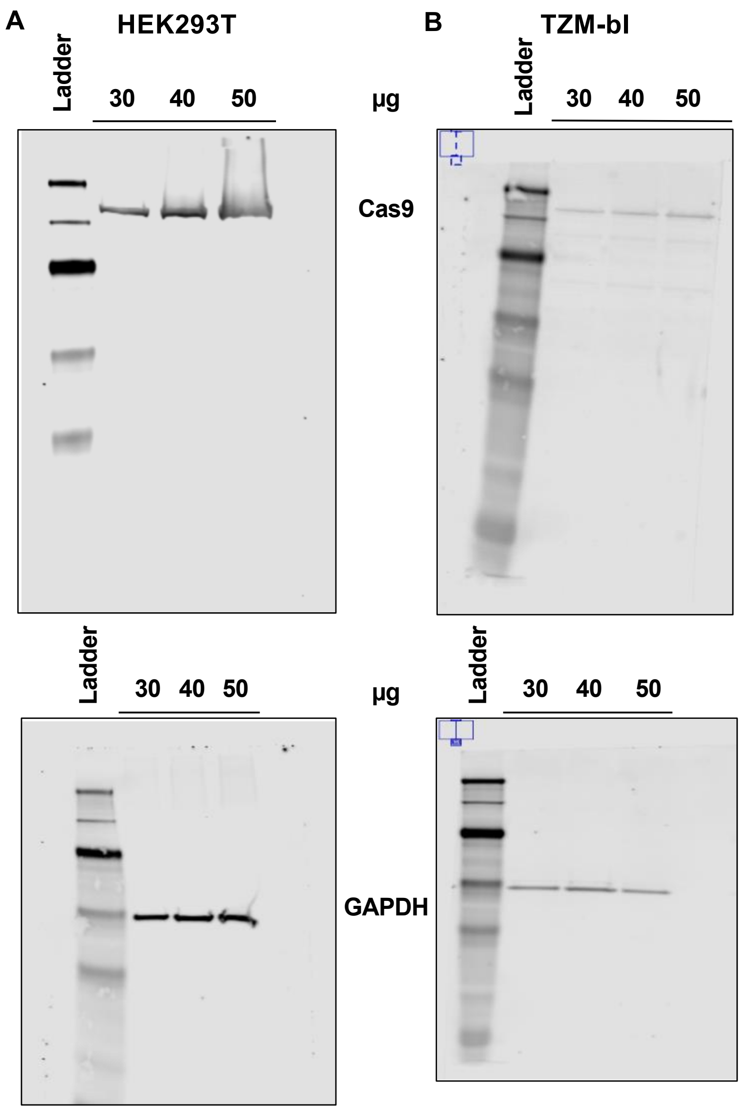

Figure S2: Validation of the CRISPR/Cas9 gene-editing system in selected cell lines. (A) HEK 293T cells were transiently transfected with Cas9 expression plasmids and harvested 24 hr post-transfection for protein lysates and western blot analyses. Increasing concentrations of protein lysates were run on a SDS-PAGE gel to demonstrate detection of Cas9 protein with a wide range of expression. GAPDH was used as a loading control. **(B)** TZM-bl cells were also transiently

transfected with Cas9 expression plasmids for downstream experimentation. Western immunoblots were performed the same as for HEK293T cells.

Figure S3

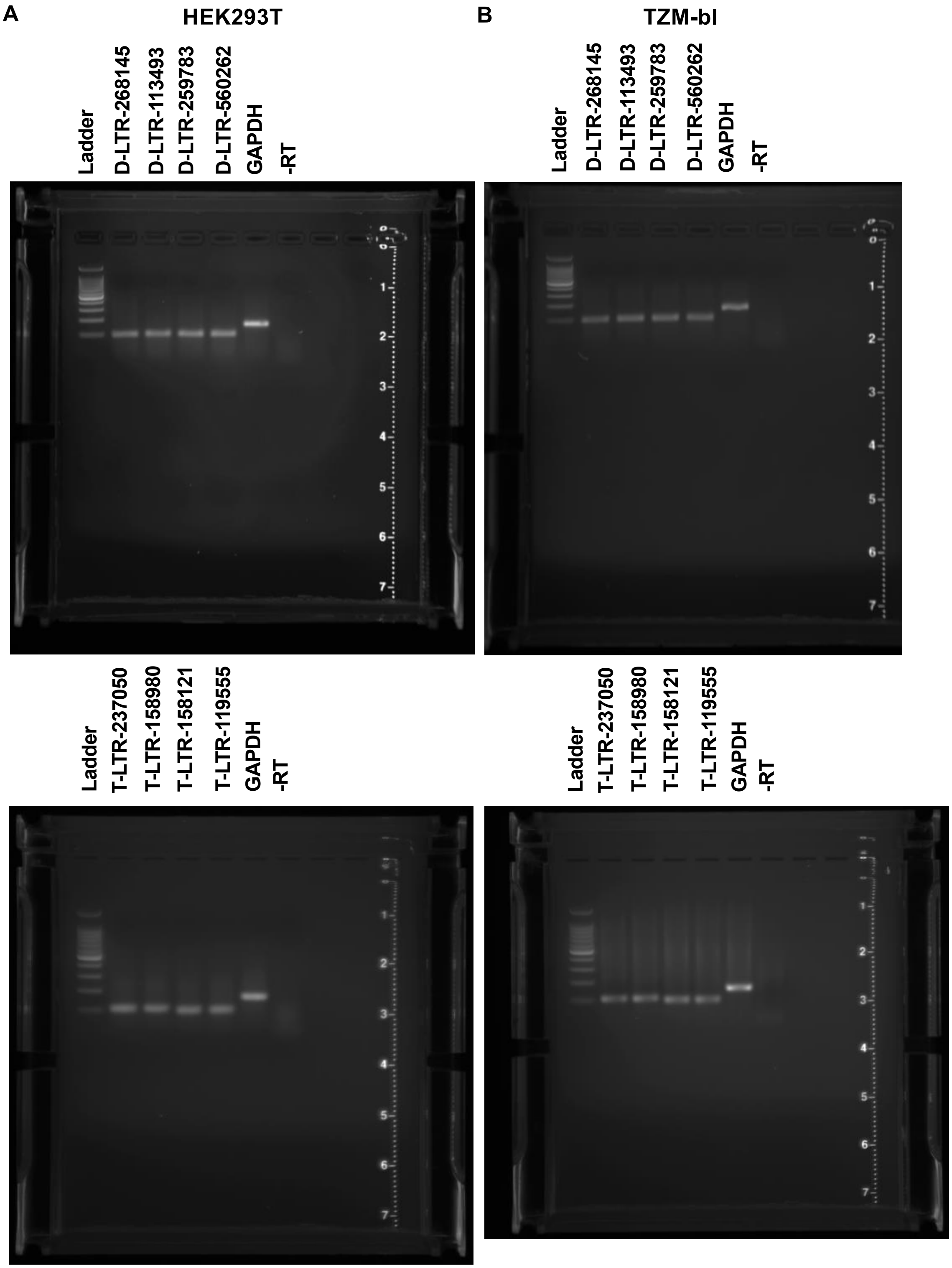
A
HEK293T
B
TZM-bl
Ladder
D-LTR-268145
D-LTR-113493
D-LTR-259783
D-LTR-560262
GAPDH
-RT
Ladder
T-LTR-237050
T-LTR-158980
T-LTR-158121
T-LTR-119555
GAPDH
-RT

Figure S3: Validation of the CRISPR/Cas9 gene-editing system in selected cell lines. (A) HEK 293T cells were transiently transfected with D-LTR-268145, D-LTR-113493, D-LTR-259783 and D-LTR-560262 and T-LTR-237050, T-LTR-158980, T-LTR-158121, and T-LTR-119555 gRNA expression plasmids for RNA isolation and RT-PCR. RT-PCR analysis demonstrated gRNA expression compared to GAPDH control. No bands were detected in the no reverse transcriptase control (-RT) lane. **(B)** TZM-bl cells were also transiently transfected with gRNA expression plasmids for downstream experimentation. RT-PCR was performed the same as for HEK293T cells.

Figure S4

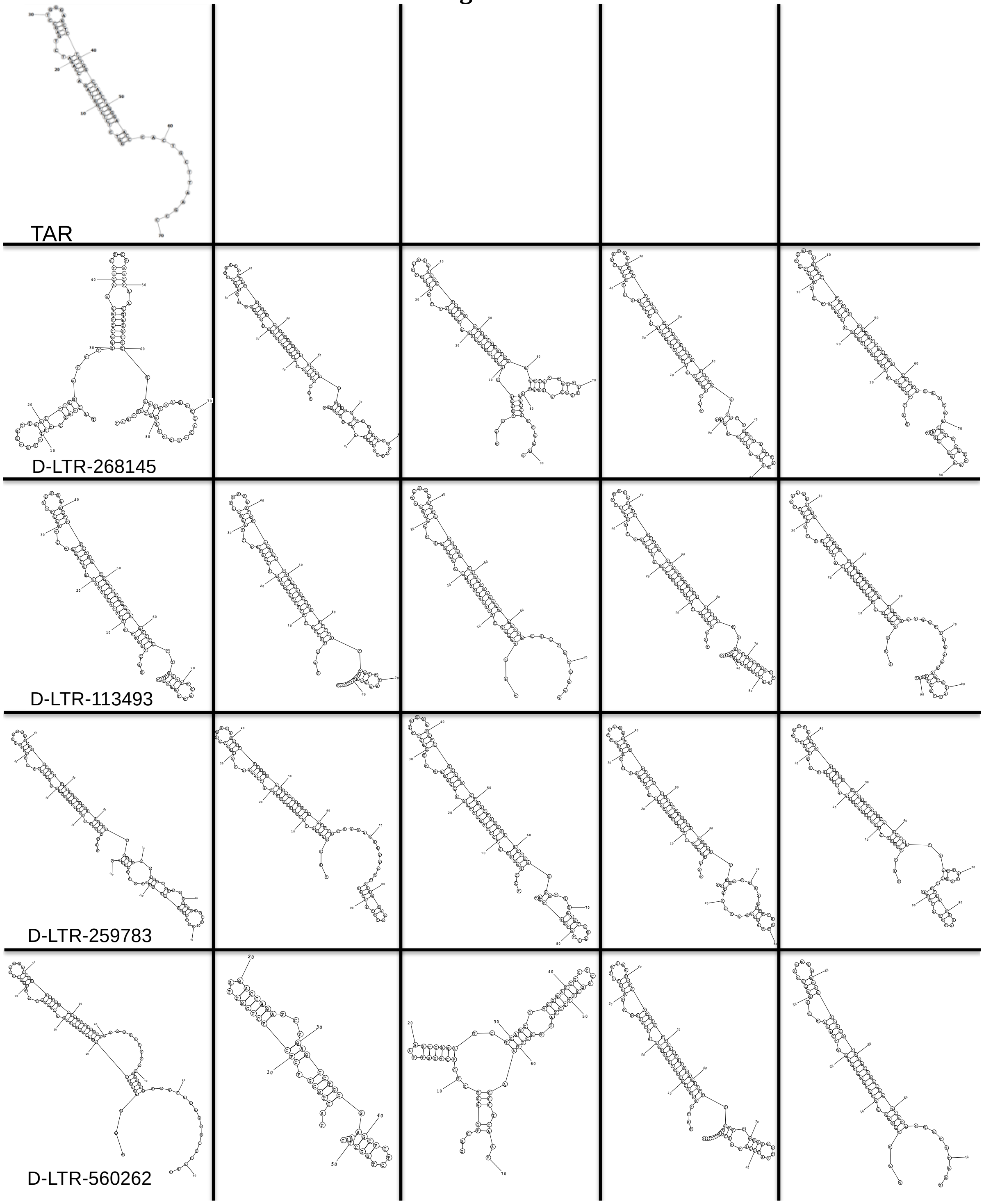

Figure S4: Five additional randomly selected *in silico* secondary structures of the TAR stem-loop when treated with Drexel gRNAs. Of these structures, you can see there is much diversity in the TAR stem loop after being treated with the Drexel gRNAs.

Figure S5

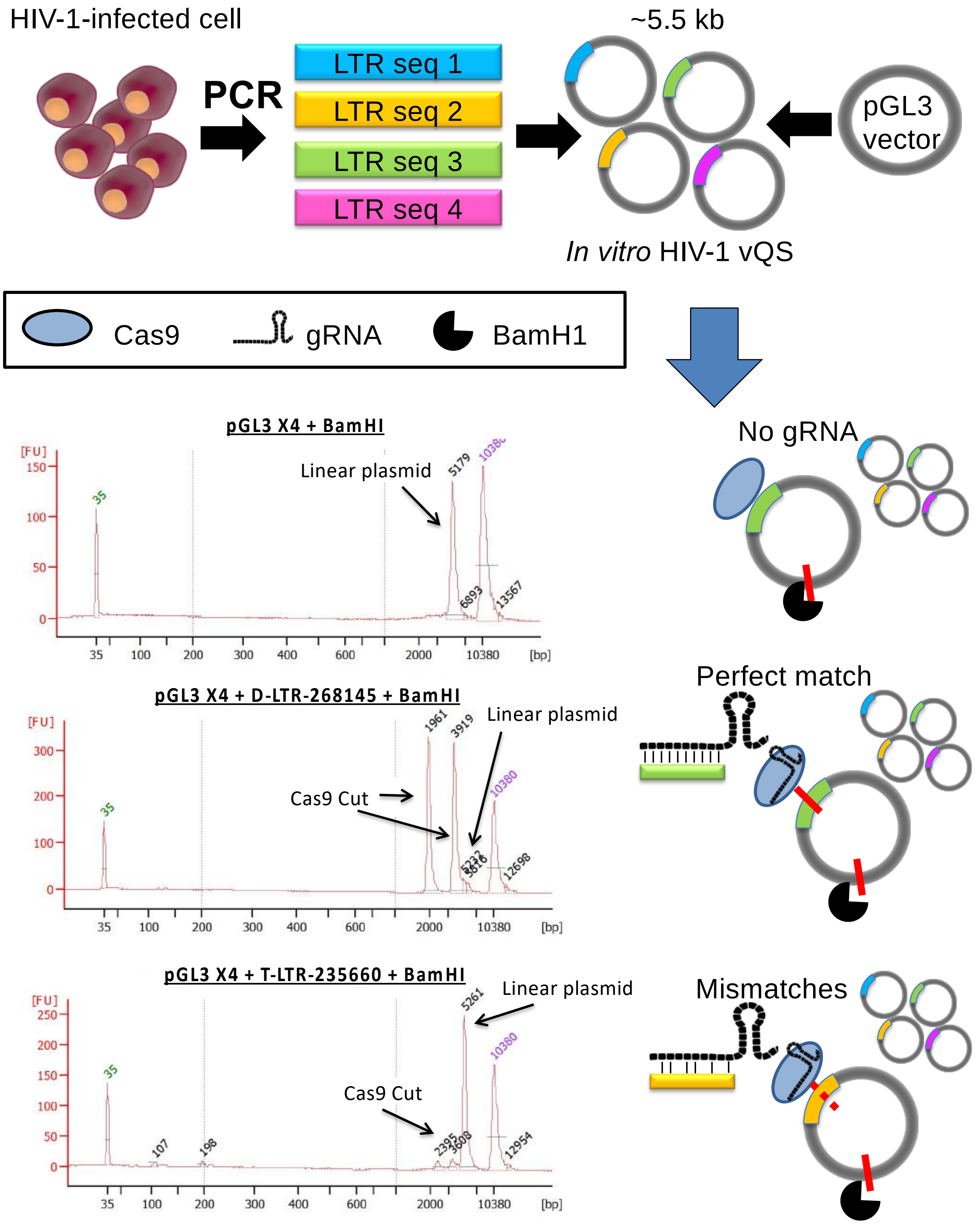

Figure S5: Schematic overview of *in vitro* cleavage assay for patient-derived HIV-1 vQS. HIV-1-infected patient PBMCs have their genomic DNA isolated and LTRs PCR amplified. The amplified LTRs were then cloned into the pGL3 vector and exposed to CAS9 and BamHI alone

or in combination with a gRNA. An example of the bioanalyzer plot for no gRNA, a perfect match gRNA and one with mismatches is shown.

Figure S6

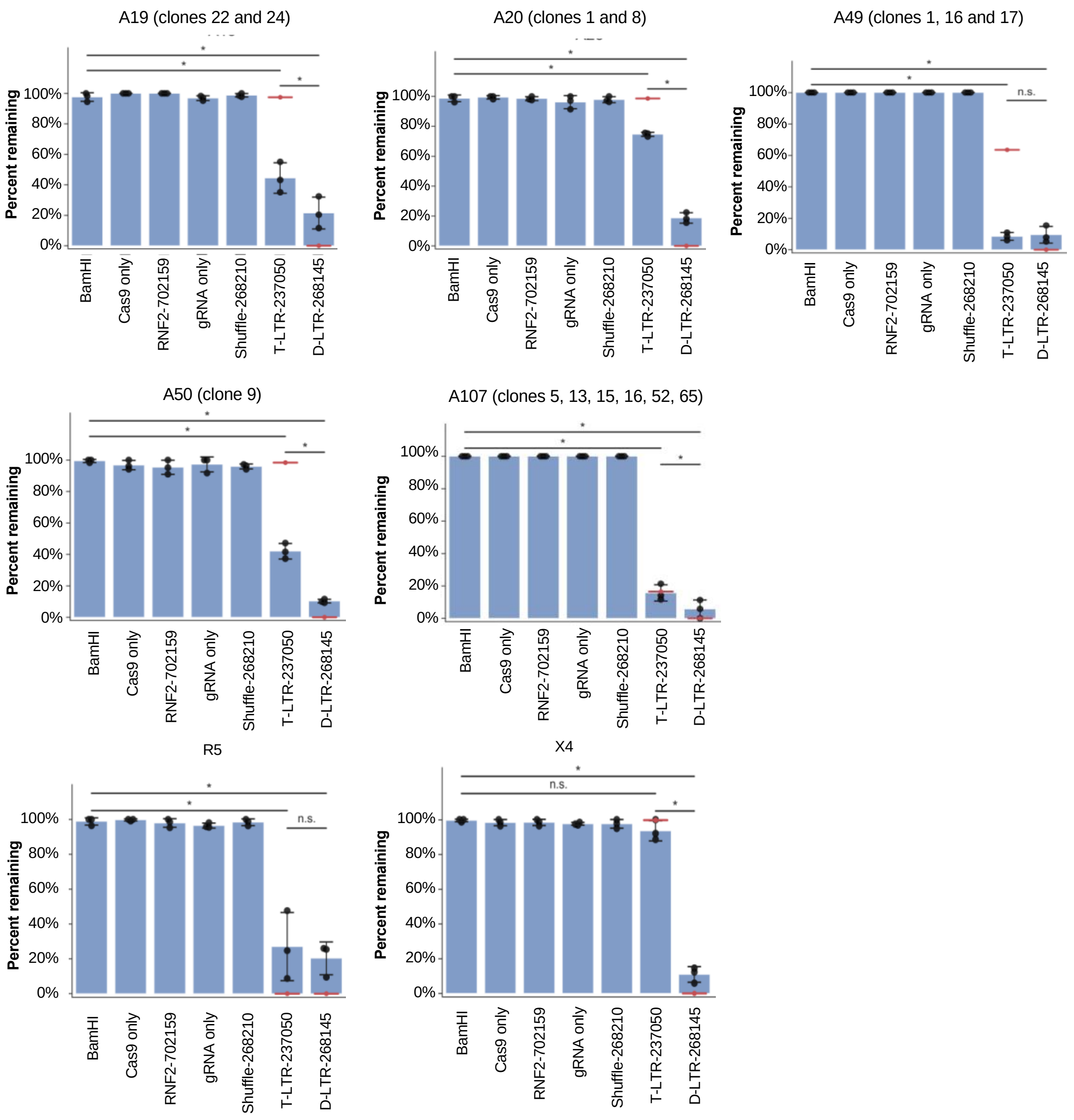

Figure S6: *In vitro* CRISPR/Cas9 cleavage assay for each of the five individual patients, X4 and R5 consensus LTRs. The figures indicate the average of three independent *in vitro* cleavage assays for each of the five patient LTRs or consensus X4 or R5 LTRs. For each patient, there was a single target or multiple targets, as indicated by the number of clones. The error bars represent the standard error of the mean. The predicted percent remaining (red bar) is the value indicated

from the value provide by the MIT tool as the likelihood to cleave a particular sequence.

Figure S7

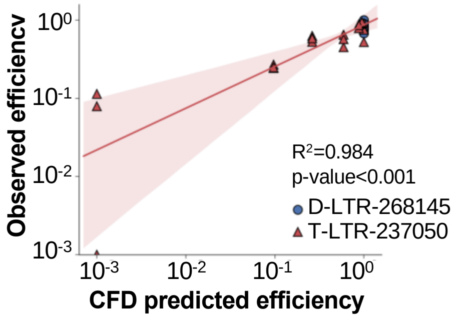

Figure S7: CFD score accurately predicts in vitro cleavage frequencies of vQS. The scatter plot represents the correlation of the observed percent cleaved in the *in vitro* assay shown in Figure 6B versus the predicted cleavage from the CFD activity score.

Table S1

Primer/Oligonucleotide Name	Sequence (5' to 3')	Techniques
LTR 3	TGGAAGGGCTAATTTATCC	Conventional PCR
LTR 4	TGACTAAAAGGGTCTGAGGG	Conventional PCR
LTR 5	CACTCCCAACGAAGACAAGA	Conventional PCR, Sequencing
LTR 6	GAGGGATCTCTAGTTACCAG	Conventional PCR, Sequencing
LTR 7	TCCTCCATCTGTGGGTCTACCA	Sequencing
GAPDH For	CAATGACCCCTTCATTGACC	RT-PCR
GAPDH Rev	TTGATTTTGGAGGGATCTCG	RT-PCR
T-LTR-237050	AGGGCCAGGGATCAGATATCCACTGACCTT	RT-PCR
T-LTR-158980	GAGTACTTCAAGAACTGCTGACATCGAGCT	RT-PCR
T-LTR-158121	GATTGGCAGAACTACACACC	RT-PCR
T-LTR-119555	GCGTGGCCTGGGCGGGACTG	RT-PCR
D-LTR-268145	TTTATTGAGGCTTAAGCAGT	RT-PCR
D-LTR-113493	CTTTATTGAGGCTTAAGCAG	RT-PCR
D-LTR-259783	ACTCAAGGCAAGCTTTATTG	RT-PCR
D-LTR-560262	ATCTGAGCCTGGGAGCTCTC	RT-PCR
gRNA seq Rev primer	CCGACTCGGTGCCACTTTTT	RT-PCR
M13 Reverse	CAGGAAACAGCTATGAC	Sequencing
T-LTR-237050	TTCTAATACGACTCACTATAGATCAGATATCCACTGACCTTGTTTTAGAGCTAGA	In vitro transcription
D-LTR-268145	TTCTAATACGACTCACTATAGTTTATTGAGGCTTAAGCAGTGTTTTAGAGCTAGA	In vitro transcription
RNF2-702159	TTCTAATACGACTCACTATAGTCATCTTAGTCATTACCTGGTTTTAGAGCTAGA	In vitro transcription
Shuffle-268210	TTCTAATACGACTCACTATAGAAGACCTATTATTACGACCAGTTTTAGAGCTAGA	In vitro transcription

Table S1: Primers and oligonucleotides used in selected methods as indicated.

Table S2

ID	gRNA Sequence	Start	Stop	Input for md5
T-LTR-237050	AGGGCCAGGGATCAGATATCCACTGACCTT.NGG	98	127	AGGGCCAGGGATCAGATATCCACTGACCTTNGG
T-LTR-158980	CCN.GAGTACTTCAAGAACTGCTGACATCGAGCT	341	312	AGCTCGATGTCAGCAGTTCTTGAAGTACTCNGG
T-LTR-158121	GATTGGCAGAACTACACACC.NGG	78	97	GATTGGCAGAACTACACACCNGG
T-LTR-119555	GCGTGGCCTGGGCGGGACTG.NGG	380	399	GCGTGGCCTGGGCGGGACTGNGG
D-LTR-268145	CCN.ACTGCTTAAGCCTCAATAAA	532	513	TTTATTGAGGCTTAAGCAGTNGG
D-LTR-113493	CCN.CTGCTTAAGCCTCAATAAAG	533	514	CTTTATTGAGGCTTAAGCAGNGG
D-LTR-259783	CCN.CAATAAAGCTTGCCTTGAGT	545	526	ACTCAAGGCAAGCTTTATTGNGG
D-LTR-560262	ATCTGAGCCTGGGAGCTCTC.NGG	475	494	ATCTGAGCCTGGGAGCTCTCNGG
D-LTR-796533	CCN.GGGAGCTCTCTGGCTAACTA	485	504	TAGTTAGCCAGAGAGCTCCCNGG
D-LTR-338488	GGGAGCTCTCTGGCTAACTA.NGG	485	504	GGGAGCTCTCTGGCTAACTANGG
D-LTR-603056	TGGGAGCTCTCTGGCTAACT.NGG	484	503	TGGGAGCTCTCTGGCTAACTNGG
D-LTR-273814	CCN.GATCTGAGCCTGGGAGCTCT	493	474	AGAGCTCCCAGGCTCAGATCNGG
D-LTR-327782	CAGAACTACACACCAGGGCC.NGG	84	103	CAGAACTACACACCAGGGCCNGG
D-LTR-145649	AGAACTACACACCAGGGCCA.NGG	85	104	AGAACTACACACCAGGGCCANGG
Shuffle-268210	AAGACCTATTATTACGACCA.NGG	-	-	AAGACCTATTATTACGACCANGG
D-LTR-P4-227913	CCN.ACTGCTTAAGCCTCAATAAA, CCN.CTGCTTAAGCCTCAATAAAG,<br>CCN.CAATAAAGCTTGCCTTGAGT, ATCTGAGCCTGGGAGCTCTC.NGG	475	545	ACTCAAGGCAAGCTTTATTGNGGATCTGAGCCTG<br>GGAGCTCTCNGGCTTTATTGAGGCTTAAGCAGNG<br>GTTTATTGAGGCTTAAGCAGTNGG
D-LTR-P10-287206	CCN.ACTGCTTAAGCCTCAATAAA, CCN.CTGCTTAAGCCTCAATAAAG,<br>CCN.CAATAAAGCTTGCCTTGAGT, ATCTGAGCCTGGGAGCTCTC.NGG,<br>CCN.GGGAGCTCTCTGGCTAACTA, GGGAGCTCTCTGGCTAACTA.NGG,<br>TGGGAGCTCTCTGGCTAACT.NGG, CCN.GATCTGAGCCTGGGAGCTCT,<br>CAGAACTACACACCAGGGCC.NGG, AGAACTACACACCAGGGCCA.NGG	84	545	ACTCAAGGCAAGCTTTATTGNGGAGAACTACACA<br>CCAGGGCCANGGAGAGCTCCCAGGCTCAGATCNG<br>GATCTGAGCCTGGGAGCTCTCNGGCAGAACTACA<br>CACCAGGGCCNGGCTTTATTGAGGCTTAAGCAGN<br>GGGGGAGCTCTCTGGCTAACTANGGTAGTTAGCC<br>AGAGAGCTCCCNGGTGGGAGCTCTCTGGCTAACT<br>NGGTTTATTGAGGCTTAAGCAGTNGG
T-LTR-P2-185220	AGGGCCAGGGATCAGATATCCACTGACCTT.NGG,<br>CCN.GAGTACTTCAAGAACTGCTGACATCGAGCT	94	341	AGCTCGATGTCAGCAGTTCTTGAAGTACTCNGGA<br>GGGCCAGGGATCAGATATCCACTGACCTTNGG
RNF2-702159	TCATCTTAGTCATTACCTGG.NGG	-	-	TCATCTTAGTCATTACCTGGNGG

Table S2: gRNA sequence, start and stop position within the HIV-1 LTR, and input for md5 hashsum number generation.
